# Discontinuation of anti-VEGF cancer therapy promotes metastasis through a liver revascularization mechanism

**DOI:** 10.1038/ncomms12680

**Published:** 2016-09-01

**Authors:** Yunlong Yang, Yin Zhang, Hideki Iwamoto, Kayoko Hosaka, Takahiro Seki, Patrik Andersson, Sharon Lim, Carina Fischer, Masaki Nakamura, Mitsuhiko Abe, Renhai Cao, Peter Vilhelm Skov, Fang Chen, Xiaoyun Chen, Yongtian Lu, Guohui Nie, Yihai Cao

**Affiliations:** 1 Key Laboratory of International Collaborations, Second People's Hospital of Shenzhen, First Affiliated Hospital of Shenzhen University, Shenzhen 518035, China; 2 Department of Microbiology, Tumor and Cell Biology, Karolinska Institute, 171 77 Stockholm, Sweden; 3 Section for Aquaculture, The North Sea Research Centre, DTU Aqua, Technical University of Denmark Hirtshals 1019850, Denmark; 4 The First Affiliated Hospital of Zhejiang Chinese Medicine University, 54 Youdian Road, Hangzhou, Zhejiang 310006, China; 5 State Key Laboratory of Ophthalmology, Zhongshan Ophthalmic Center, Sun Yat-Sen University, Guangzhou 510060, China; 6 Department of Cardiovascular Sciences, University of Leicester and NIHR Leicester Cardiovascular Biomedical Research Unit, Glenfield Hospital, Leicester LE3 9QP, UK

## Abstract

The impact of discontinuation of anti-VEGF cancer therapy in promoting cancer metastasis is unknown. Here we show discontinuation of anti-VEGF treatment creates a time-window of profound structural changes of liver sinusoidal vasculatures, exhibiting hyper-permeability and enlarged open-pore sizes of the fenestrated endothelium and loss of VE-cadherin. The drug cessation caused highly leaky hepatic vasculatures permit tumour cell intravasation and extravasation. Discontinuation of an anti-VEGF antibody-based drug and sunitinib markedly promotes liver metastasis. Mechanistically, host hepatocyte, but not tumour cell-derived vascular endothelial growth factor (VEGF), is responsible for cancer metastasis. Deletion of hepatocyte VEGF markedly ablates the ‘off-drug'-induced metastasis. These findings provide mechanistic insights on anti-VEGF cessation-induced metastasis and raise a new challenge for uninterrupted and sustained antiangiogenic therapy for treatment of human cancers.

## Abstract (web summary)

Anti-VEGF therapy often produces limited beneficial effects in cancer patients. Here, the authors show that discontinuation of anti-VEGF cancer therapy in xenografts-bearing mice increases cancer cells extravasation and intravasation in liver through the host-derived VEGF.

## Graphical abstract

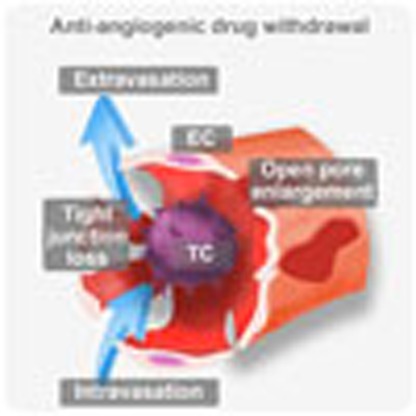

Anti-VEGF-based antiangiogenic drugs including bevacizumab, aflibercept, ramucirumab and tyrosine kinase inhibitors (TKIs) targeting vascular endothelial growth factors (VEGFRs) are routinely used for treatment of various human cancers^1^,^2^,^3^,^4^,^5^,^6^,^7^,^8^,^9^. In general, survival improvement by addition of an antiangiogenic component to conventional chemotherapeutics is modest^2^,^3^,^10^,^11^,^12^. For example, multiple lines of clinical trials have shown that treatment of colorectal cancer (CRC) patients with bevacizumab only produced very limited beneficial effects^4^,^13^,^14^,^15^,^16^,^17^,^18^. At this time of writing, it is still unclear about the fundamental mechanism by which these anti-VEGF drugs in combination of chemotherapy produce clinical benefits. Moreover, most rigorous clinical trials demonstrate that anti-VEGF monotherapy rarely improves overall survivals of patients with most cancer types^4^,^6^,^7^,^8^,^9^,^10^,^11^,^12^. The lack of sufficient clinical benefits of anti-VEGF drugs in human cancer patients has raised several unresolved issues including: the mode of action, selection of responsive patient populations, treatment timeline and drug resistance mechanisms. Based on a series of preclinical and clinical studies, many speculative hypotheses and concepts have been put forward to explain these unresolved clinical issues. It is generally believed that blocking the VEGF-VEGFR signalling would augment compensatory mechanisms of tumour angiogenesis by elevating expression levels of angiogenic factors that these drugs do not target, circumventing the VEGF-dependent angiogenic signals^2^,^19^,^20^,^21^,^22^. Another hypothesis claims that anti-VEGF drugs normalize tumour vasculatures and blood perfusion, and alleviate tumour hypoxia, leading to improved delivery of chemotherapeutics in combination therapeutic settings^23^. For intrinsic non-responders, it is speculated that tumours employ non-VEGF proangiogenic factors to grow vessels. However, these hypotheses warrant further clinical validation.

VEGF (also called VEGF-A) is the prototype of a family of angiogenic factors and it modulates angiogenesis, vascular remodelling, vascular permeability and multiple non-vascular functions^24^,^25^,^26^. For high-affinity binding receptors, endothelial VEGFR2 transduces angiogenic and vascular permeability signals, whereas VEGFR1 might commit to some non-vascular functions or serve as a decoy signalling system^27^,^28^. Almost all human tumour tissues express high levels of VEGF relative to their healthy tissue counterparts^29^. Thus, various drugs targeting the VEGF-VEGFR signalling pathway have been developed for treatment of human cancer patients. Intervention at almost every step of the VEGF signalling pathway has been considered for drug development. For example, bevacizumab neutralizes VEGF, ramucirumab binds to VEGFR2 and blocks its interaction with VEGF, aflibercept traps VEGF ligands, TKIs block activation of VEGFRs, and everolimus, temsirolimus and zotarolimus inhibit the downstream mechanistic target of rapamycin functions^24^. In addition to clinical benefits, the antiangiogenic therapy-altered tumour microenvironment has been cautioned for promoting metastasis in experimental mouse models^30^,^31^.

Despite the fact that original designs of these drugs are targeting the tumour vasculature, during clinical practice, anti-VEGF drugs are systemically delivered to cancer patients. To date, no available antiangiogenic drugs are specifically delivered to the tumour local microenvironment. Systemic administration of anti-VEGF agents would indistinguishably cause global drug exposure to all tissues and organs. Recent studies from our laboratory and others show that systemic delivery of anti-VEGF drugs produces broad effects on regression of healthy vasculatures in various organs^32^,^33^. In addition, there has been lacking a unified opinion on timeline of antiangiogenic therapy. In theory, non-stop treatment with anti-VEGF drugs should be given to cancer patients as VEGF continues to be functional after discontinuation of treatment. However, during clinical practice, interrupted anti-VEGF regimens are used in cancer patients because of drug-related adverse effects, economic reasons or drug resistance. It is unclear if withdrawal of antiangiogenic therapy would produce harmful effects that may jeopardize patient survivals. This important issue has not been fully explored although discontinuation of VEGF treatment is routinely undertaken during clinical practice. In particular, the ‘off-drug'-associated vascular changes of non-tumour healthy vasculatures in various tissues and organs in promoting cancer metastasis are completely unknown.

In the present work, we have studied discontinuation of anti-VEGF therapy-altered healthy hepatic vasculatures in facilitating cancer metastasis. In several mouse tumour models, we have validated the concept that the anti-VEGF cessation-associated regrowth and remodelling of hepatic vasculatures provide a structural basis of cancer metastasis. Mechanistically, the host hepatocyte- but not tumour cell-derived VEGF is responsible for facilitating cancer metastasis. Based on these findings, non-stop persistent anti-VEGF therapy is recommended for treatment of human cancer patients and cautions should be paid during drug holidays.

## Results

### Liver vascular changes by on- and off-anti-VEGF therapy

**Figure 1:**
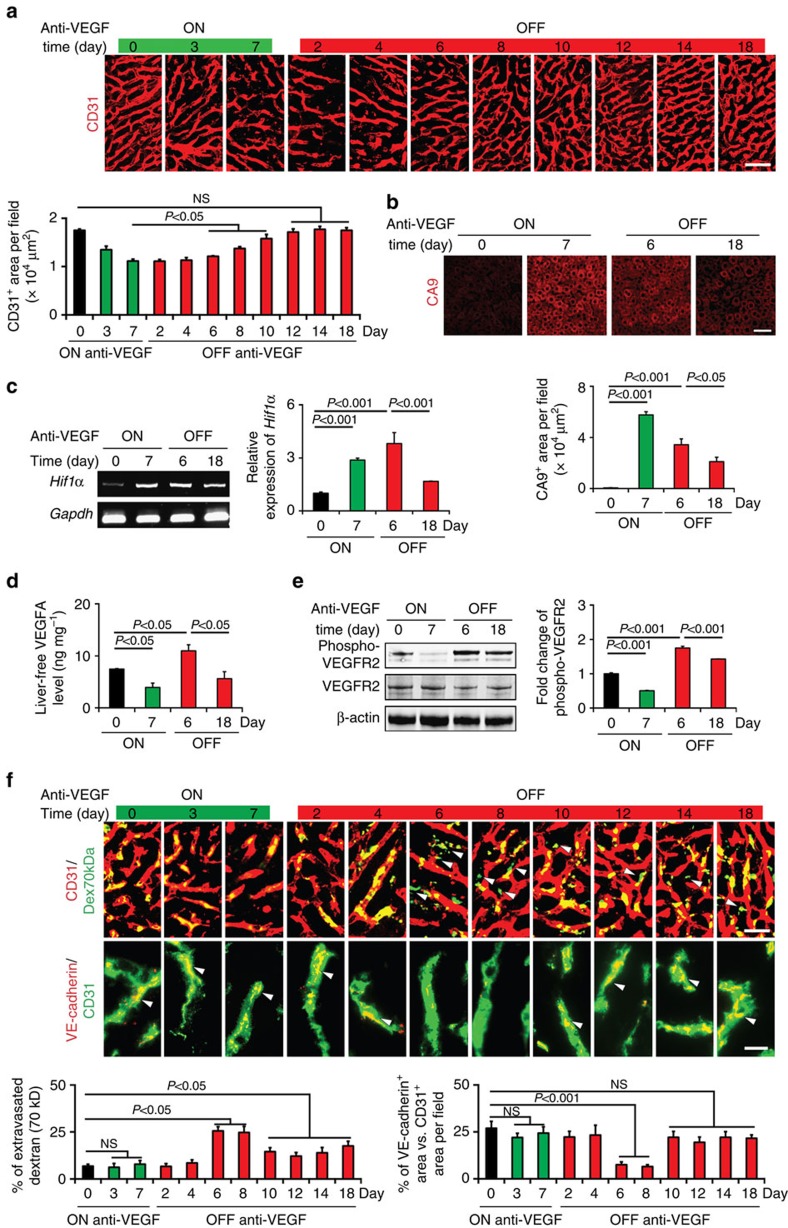
Revascularization of liver microvasculatures after withdrawal of systemic anti-VEGF treatment. (**a**) Time-course analysis of CD31^+^ microvessels in livers before anti-VEGF treatment, on-drug and off-drug. Scale bar, 50 μm. Data were quantified from nine random fields per group. (**b**) Liver tissue hypoxia measured by CA9 expression in various groups. Scale bar, 100 μm. Data were quantified from nine random fields per group. (**c**) RT-PCR and qPCR quantification of *Hif1a* expression in various on- and off-drug groups (triplicates per group). (**d**) ELISA measurement of non-antibody-bound free VEGF in different groups (triplicates per group). (**e**) Measurement and quantification of total and phosphorylated VEGFR2 protein levels in various groups (triplicates per group). (**f**) Measurements of extravasation of Fluorescein-labelled 70-kDa-dextran and expression levels of VE-cadherin in different groups. Arrows point to leaked dextran signals. Scale bar in upper panel, 25 μm. Scale bar in lower panel, 10 μm. Data were quantified from nine random fields per group. OFF, off-drug; ON, on-drug. (mean±s.e.m., NS, not significant, Student's *t*-test); Full gel images for **e** are shown in Supplementary Fig. 8.

Liver metastasis is commonly seen in patients with various cancers. In particular, CRC often metastasizes to liver and bevacizumab in combination with chemotherapeutics is given as the first-line option for treatment of CRC patients^4^. To study the impact of anti-VEGF treatment on hepatic vasculature, a rabbit anti-mouse VEGF neutralizing antibody (VEGF blockade)^32^,^34^,^35^,^36^,^37^ was used for treatment of tumour-free healthy mice (Supplementary Fig. 1a). After receiving VEGF blockade for 7 days, marked regression of hepatic vasculatures was observed (Fig. 1a). Discontinuation of VEGF blockade resulted in a rapid revascularization of hepatic vasculatures despite the long half-life of VEGF blockade^36^. By day 12, hepatic vessels recovered to the non-treated level (Fig. 1a). During recovery angiogenesis, hepatic vasculatures did not exhibit obvious disorganization and were functionally perfused (Fig. 1a and Supplementary Fig. 1b). Hepatic vasculatures exhibited positivity of fibronectin and collagen IV, two principal components of the extracellular matrix and basement membrane around hepatic microvessels (Supplementary Fig. 1c). VEGF blockade had no effects on fibronectin and collagen IV expression. Notably, cessation of VEGF blockade-induced revascularization occurred along the trails of the basement membrane sleeves (Supplementary Fig. 1c). Hepatic microvessels lacked laminin and NG2^+^ pericytes, except the central arterial vessel (Supplementary Fig. 1d).

Without VEGF blockade treatment, healthy liver tissue experienced very modest hypoxia as measured by CA9 levels and VEGF blockade augmented severe tissue hypoxia even after cessation of treatment for 18 days (Fig. 1b). Consistently, HIF-1α expression levels were elevated during VEGF blockade treatment (Fig. 1c). Consistent with elevated hypoxia and HIF-1α expression levels, liver VEGF mRNA and the non-antibody-bound free VEGF protein levels were also significantly increased at day 6 after withdrawal of VEGF blockade (Fig. 1d and Supplementary Fig. 1e). The mechanism underlying hypoxia-induced increased Hif-1α mRNA level might be due to increased stability of Hif-1α mRNA. Liver non-antibody-bound free VEGF molecules were able to activate VEGFR2 by stimulation of phosphorylation (Fig. 1e). The phosphorylated VEGFR2 levels corresponded with the elevated levels of liver-free VEGF protein. Similar hepatic vascular changes in response to on- and off-anti-VEGF therapy were also seen in tumour-bearing mice (Supplementary Fig. 1f)

Time-course studies showed that revascularization of hepatic vasculatures exhibited hyper-permeability of 70-kDa-dextran between days 6 and 8 after cessation of VEGF blockade treatment (Fig. 1f). Increases of vascular leakage persisted for at least 18 days after discontinuation of anti-VEGF treatment, albeit the levels of leakiness were weakened relative to days 6 and 8 (Fig. 1f). Reconciling with hyper-vascular leakiness, regenerated hepatic vessels showed transient loss of VE-cadherin, an adhering protein zipping endothelial cell tight junctions. After day 10 off-drug, VE-cadherin expression levels recovered to the untreated physiological level (Fig. 1f).

To further validate these findings, we used a clinically available TKI, sunitinib, for treatment of tumour-free healthy mice. Sunitinib treatment in principle reproduced similar results as the anti-VEGF neutralizing antibody (Fig. 1 and Supplementary Figs 1–3). There were few particular characteristics of sunitinib that distinguished from the anti-VEGF antibody treatment. First, the off-drug-triggered revascularization occurred much faster than VEGF blockade. After only day 6 off-drug, revascularization of hepatic vessels reached to the untreated physiological level (Supplementary Fig. 2). The off-drug-triggered rapid revascularization likely reflected the short half-life of sunitinib as a small molecule. The second interesting feature was that liver VEGF protein level was markedly elevated during sunitinib treatment, which persisted at least until day 2 off-drug (Supplementary Fig. 2d). Despite the elevated hepatic VEGF level at day 7 during treatment, VEGFR2 in the liver tissue did not become significantly phosphorylated (Supplementary Fig. 2e). Robust VEGFR2 phosphorylation was only detected at day 2 off-drug, indicating ineffectiveness of sunitinib and the presence of high level of VEGF in the liver tissue (Supplementary Fig. 2e). Third, increased vascular leakage was found during sunitinib treatment, probably reflecting the short half-life of this small molecule (Supplementary Fig. 2f).

### Off-drug induces enlargement of pore sizes of liver vessels

**Figure 2:**
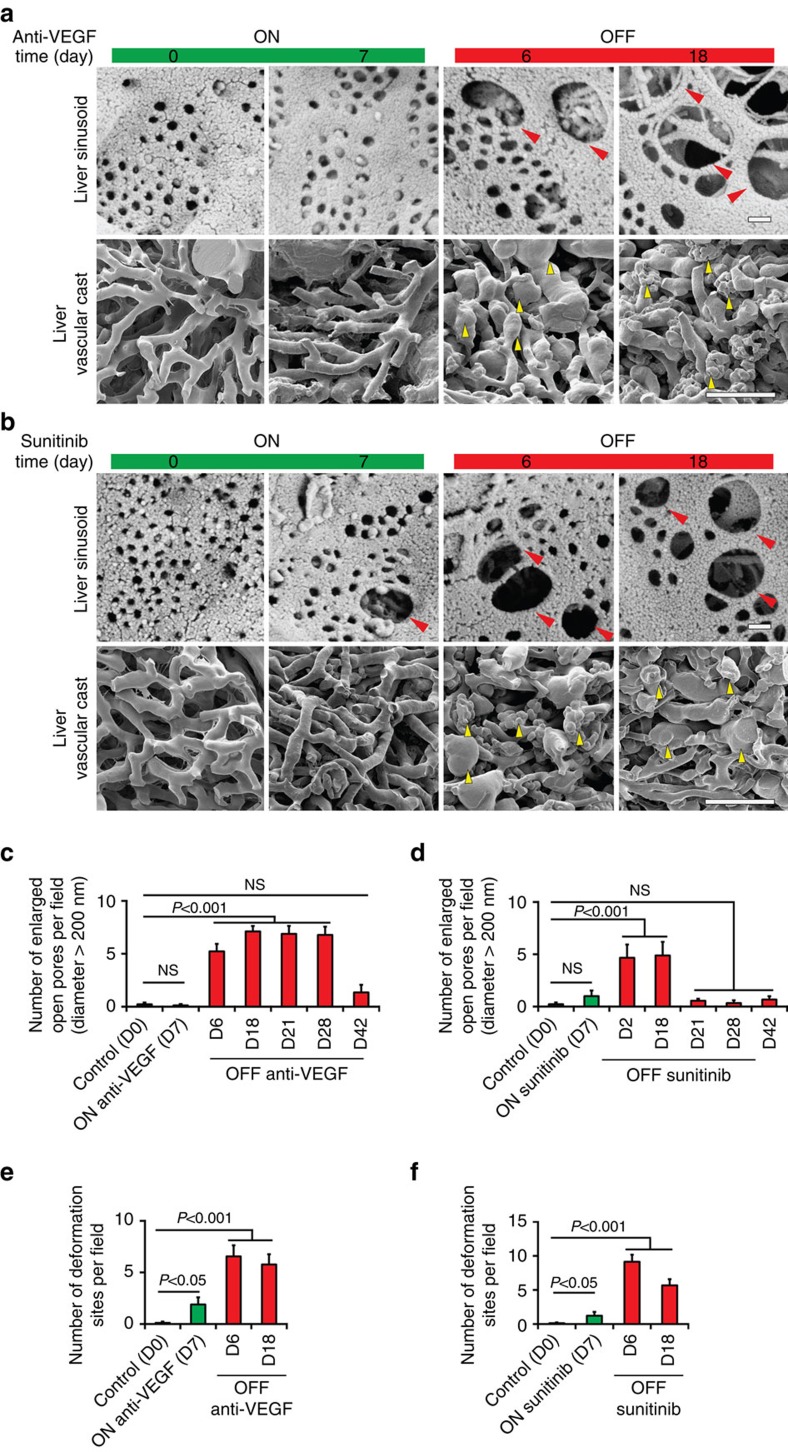
Scanning electron microscopic and vascular cast analyses of liver microvasculatures. (**a**,**b**) Representative scanning electron microscopy and vascular cast micrographs of liver microvessels of various groups. Red arrowheads point to enlarged open pores of the liver sinusoidal endothelium. Yellow arrowheads indicate bulb-like leaky structures of liver microvessels. Scale bar in upper panels, 200 nm. Scale bar in lower panels, 50 μm. (**c**,**d**) Quantifications of numbers large open pores above 200 nm in diameters. Data were quantified from 10 random fields per group. (**e**,**f**) Quantifications of numbers bulb-like leaky structures. Data were quantified from 10 random fields per group. OFF, off-drug; ON, on-drug. (mean±s.e.m., NS, not significant, Student's *t*-test).

Under physiological condition, liver microvasculatures consist of sinusoidal discontinuous capillaries that possess incomplete basal membrane and fenestrated endothelial lining, manifesting sinusoidal open pores on the vessel wall. The average size of sinusoidal open pores in mouse liver was ∼75 nm in diameter and anti-VEGF treatment did not significantly alter fenestration pore sizes (Fig. 2a,c). Cessation of anti-VEGF blockade and sunitinib resulted in marked structural changes of liver sinusoidal capillaries with marked enlargement of pore sizes. Sinusoidal pore sizes of 15–20-fold increase were often observed after discontinuation of anti-VEGF treatments (Fig. 2a–d). For the VEGF blockade-treated liver, pore enlargement occurred at day 6 off-drug and persisted at least until day 28 after treatment (Fig. 2a,c and Supplementary Fig. 4a). At day 42 off-drug, sinusoidal pore sizes returned to those before treatment (Supplementary Fig. 4). Compared with VEGF blockade, sunitinib-treated liver showed early expansion of sinusoidal pore sizes already at day 2 off-drug (Fig. 2b,d). However, enlarged pores reversed to normal sizes already at day 21 off-drug (Supplementary Fig. 4b). Diameters of some sinusoidal pores reached 1,000 nm (Supplementary Fig. 4c). The off-sunitinib triggered early occurrence and termination of pore enlargement again reflected a short half-life relative to the VEGF blockade. Of note, vascular cast imaging analysis showed distinctive features of liver sinusoidal capillaries after discontinuation of treatment. Both off-VEGF blockade and off-sunitinib triggered bulb-like structures, reflecting highly leakiness of liver sinusoidal capillaries (Fig. 2a,b,e,f).

### Off-drug promotes metastasis by increasing extravasation

**Figure 3:**
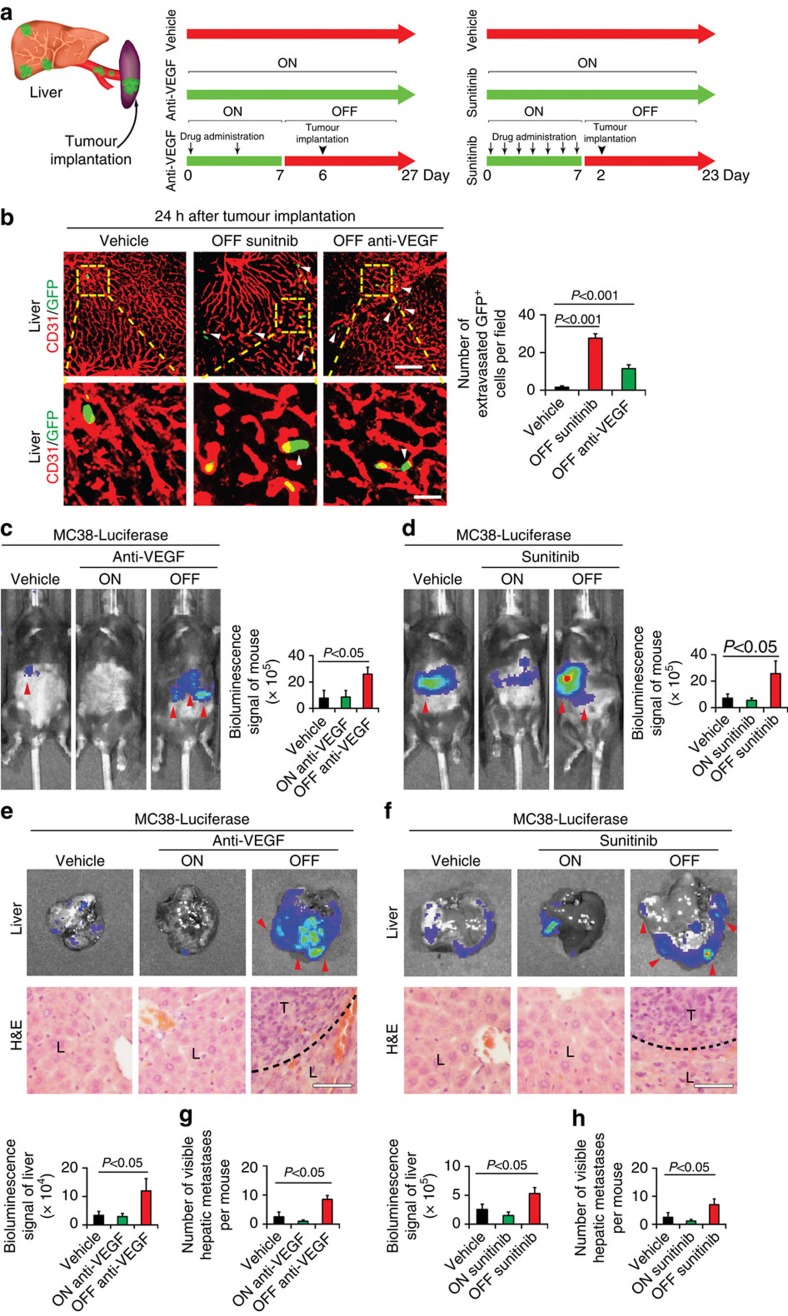
CRC tumour cell extravasation and metastasis in livers. (**a**) Schematic model of MC38 tumour cell implantation in spleen and liver metastasis and treatment schemes. (**b**) Histological analysis of extravasation of GFP^+^ tumour cells in livers received on- and off-anti-VEGF therapy. Arrowheads point to extravasated tumour cells. Scale bar in upper panel, 100 μm. Scale bar in upper panel, 25 μm. Data were quantified from nine random fields per group. (**c**,**d**) Representative mouse pictures of various groups subjected to luminescent imaging analysis of luciferase activity in metastatic cancers. Red arrowheads point to luciferase positivity. Data were quantified from 6 mice per group. (**e**,**f**). Representative liver micrographs subjected to luminescent luciferase activity analysis (*n*=6 animals per group). Red arrowheads point to luciferase positivity. H&E histological analysis of liver metastasis. Dashed lines mark the borders between tumour and liver tissues. L, liver; T, tumour. Scale bar in lower panels, 25 μm. Quantifications of liver luciferase activity (*n*=6 animals per group). (**g**,**h**) Quantifications of visible surface liver metastatic nodules (*n*=6 animals per group). OFF, off-drug; ON, on-drug. (mean±s.e.m., Student's *t*-test).

Giving the fact that withdrawal of anti-VEGF therapy induced hyper-leakiness and loss of VE-cadherin in hepatic capillaries, we hypothesized that off-drug would increase tumour cell extravasation in liver. To test this hypothesis, we chose a CRC model for two main reasons. First, liver metastasis is commonly seen in CRC patients^14^. Second, anti-VEGF treatment with bevacizumab has been approved as the key component of the first-line therapy for treatment of CRC patients^4^. To study the possible increase of CRC cell extravasation, green fluorescent protein (GFP)-and luciferase-labelled mouse MC38 CRC cells were injected into the mouse spleen. This model recapitulated the clinical situation of spontaneous metastasis (Fig. 3a).

Before tumour cell implantation, animals were pretreated with VEGF blockade or sunitinib for 7 days. Of interest, marked increases of tumour cell extravasation were detected after only 24 h tumour implantation at day 6 off-VEGF blockade and at day 2 off-sunitinib (Fig. 3b). It seemed that discontinuation of sunitinib augmented a higher level of CRC cell extravasation relative to the VEGF blockade (Fig. 3b). After 3-week tumour implantation, discontinuation of anti-VEGF therapy augmented marked increases of liver metastasis as compared with the group receiving continuous anti-VEGF treatment (Fig. 3c,d). Notably, both groups of mice receiving discontinuation of VEGF blockade and sunitinib showed higher metastatic luciferase signals than their corresponding non-treated groups (Fig. 3c,d). Gross examination and histological analysis of liver tissues further validated the presence of increased numbers and sizes of metastatic nodules (Fig. 3e–h). We further investigated tumour cell extravasation in livers after 18 days off-drug. Despite complete recovery of liver microvessel density at this time point, capillaries contained enlarged open pores and exhibited high leakiness (Figs 1 and 2 and Supplementary Fig. 4). Therefore, these structural features could be accessible for tumour cell extravasation, which eventually led to increases of liver metastasis. Indeed, implantation of MC38 CRC cells in spleen at day 18 after cessation of anti-VEGF therapy resulted in significant increases of liver metastasis (Supplementary Fig. 5). These findings demonstrate that discontinuation of anti-VEGF therapy facilitates liver metastasis.

### Tumour VEGF-independent mechanism of off-drug metastasis

To exclude the possibility of tumour cell-derived VEGF in attributing liver metastasis in our off-drug settings, we used VEGF-null tumour cells that completely lacked the *Vegf* gene^37^,^38^. Consistent with genetic deletion of the *Vegf* gene, VEGF-null tumour cells completely lacked a detectable level of VEGF protein (Supplementary Fig. 6a). In the spontaneous spleen metastasis model, deletion of tumour cell VEGF did not prevent the anti-VEGF cessation-triggered liver metastasis (Supplementary Fig. 6b–e). Withdrawal of VEGF blockade and sunitinib resulted in accelerated liver metastases, which were validated by expression of luciferase expression and histological examination.

**Figure 4:**
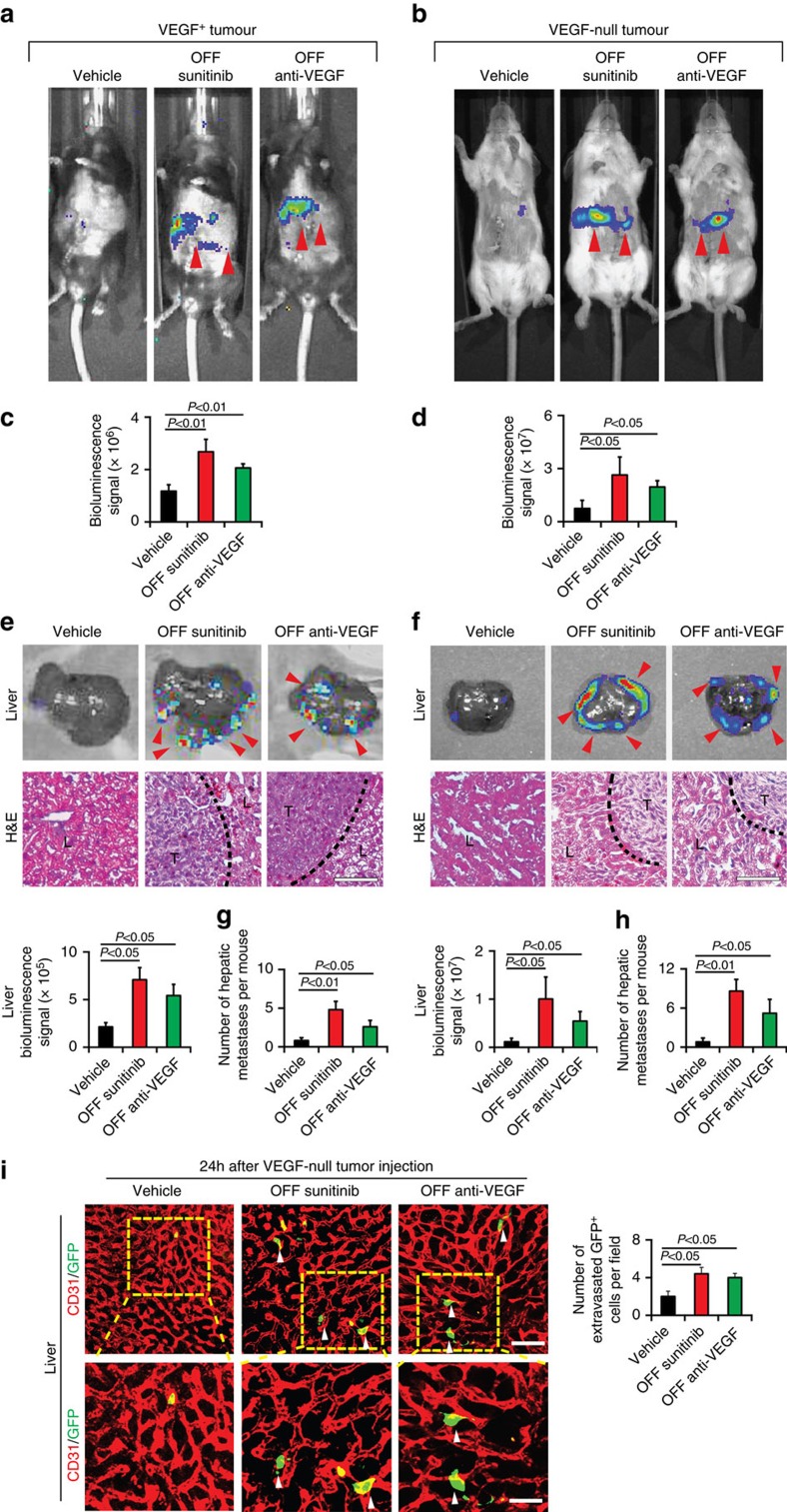
Tumour VEGF-independent liver metastasis. (**a–d**) Representative mouse pictures of various groups subjected to luminescent imaging analysis of luciferase activity in VEGF^+^ and VEGF^−^ metastatic cancers. Red arrowheads point to luciferase positivity. Data were quantified from six mice per group. (**e**,**f**) Representative liver micrographs subjected to luminescent luciferase activity analysis (*n*=6 animals per group). Red arrowheads point to luciferase positivity. H&E histological analysis of liver metastasis. Dashed lines mark the borders between tumour and liver tissues. L, liver; T, tumour. Scale bar in lower panels, 25 μm. Quantifications of liver luciferase activity (*n*=6 animals per group). (**g**,**h**) Quantifications of visible surface liver metastatic nodules (*n*=6 animals per group). (**i**) Extravasation of VEGF-null tumour cells at 24 h after tumour cell implantation in various off-drug groups. Arrowheads point to extravasated tumour cells. Scale bar in upper panel, 50 μm. Scale bar in lower panel, 25 μm. Quantification of extravasated GFP^+^ tumour cells (*n*=8 fields per group). OFF, off-drug; ON, on-drug. (mean±s.e.m., Student's *t*-test).

To further investigate tumour cell extravasation through regenerated liver microvessels, we used an independent metastatic model by injecting tumour cells into the portal vein. Similar to the spleen model, withdrawal of VEGF blockade at day 6 and sunitinib at day 2 resulted in significant increases of liver metastasis (Fig. 4a–h). It should be emphasized that the number of metastatic nodules in off-VEGF blockade and off-sunitinib groups were markedly higher than their corresponding controls, reflecting the increases of extravasated tumour cells (Fig. 4a–h). Again, deletion of the *Vegf* gene in tumour cells did not significantly affect the enhanced liver metastasis after cessation of anti-VEGF therapy (Fig. 4a–h). Similar to the spontaneous spleen metastasis model, injection of tumour cells into the portal vein markedly increased tumour cell extravasation at day 6 off-VEGF blockade and at day 2 off-sunitinib (Fig. 4i). These results further validated off-anti-VEGF therapy-induced extravasation in a portal vein metastasis model. Thus, our results from two independent models support the fact that discontinuation of anti-VEGF therapy promotes liver metastasis through a tumour cell VEGF-independent mechanism.

### Hepatocyte VEGF facilitates metastasis

**Figure 5:**
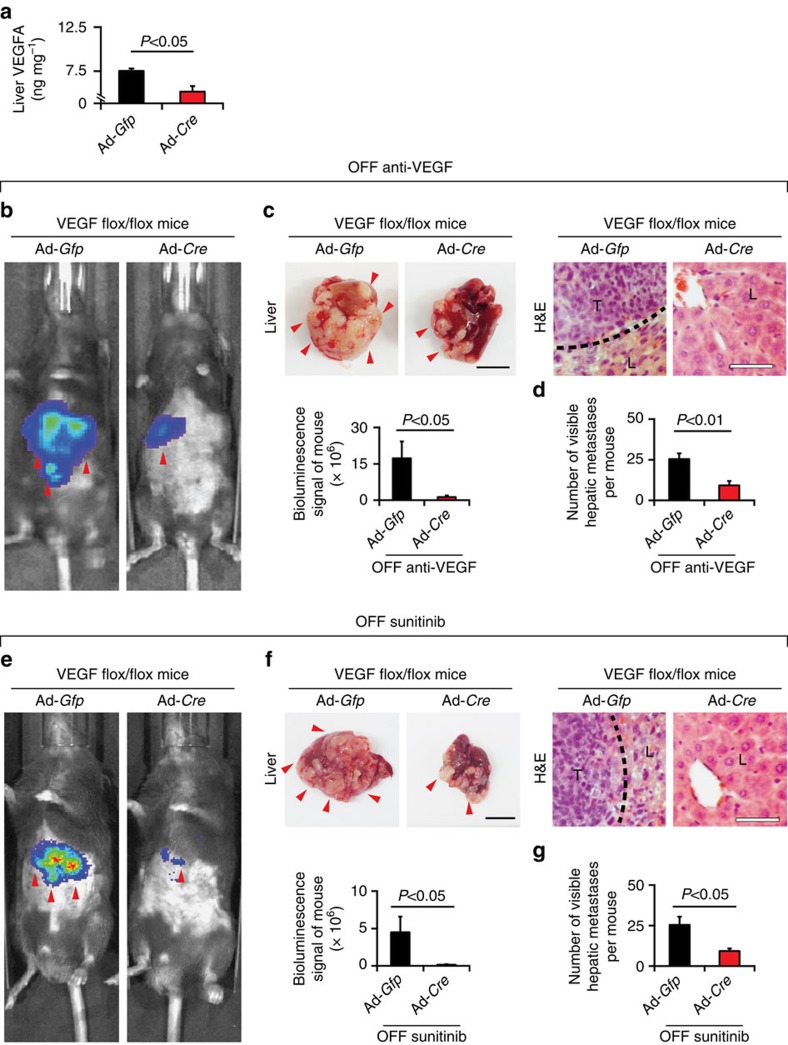
Hepatocyte-derived VEGF facilitates metastasis. *Vegf*^*flox/flox*^mice in C57bl/6 received i.v. injection with 1 × 10^9^ PFU Adenovirus-Cre viral particles per mouse (*n*=6 mice per group). (**a**) At day 3 after twice injections, liver VEGF protein levels were detected (*n*=6 mice per group). An Ad-Gfp virus was used as a control. (**b**,**e**) Representative mouse pictures of various groups subjected to luminescent imaging analysis of luciferase activity. Red arrowheads point to luciferase positivity. Data were quantified from six mice per group. (**c**,**f**) Representative liver pictures (*n*=6 animals per group). Red arrowheads point to visible metastatic nodules. H&E histological analysis of liver metastasis. Dashed lines mark the borders between tumour and liver tissues. L, liver; T, tumour. Scale bar in left panels, 1 cm. Scale bar in right panels, 25 μm. (**d**,**g**) Quantification of visible surface liver metastatic nodules (*n*=6 animals per group). OFF, off-drug; ON, on-drug. (mean±s.e.m., Student's *t*-test).

To define the molecular players attributing to the drug withdrawal-triggered liver metastasis, we further studied the role of host VEGF in promoting metastasis. Since VEGF is a key angiogenic factor in maintaining the fenestrated vascular structures in different tissues and organs, it is likely that the host-derived VEGF attributes to metastasis. The fact that discontinuation of anti-VEGF treatment increased expression levels of VEGF in the liver tissue supports this notion (Fig. 1 and Supplementary Fig. 1). To provide further experimental evidence, we took a genetic approach to delete hepatocyte VEGF by delivery of an adenovirus-Cre in *Vegf*^*flox/flox*^mice. This approach has previously been shown to effectively delete VEGF expression in hepatocytes^39^. Indeed, this conditional knockout approach effectively reduced VEGF expression hepatocyte VEGF (Fig. 5a). Along with reduction of hepatocyte VEGF levels, drug-cessation-triggered liver metastasis of MC38 CRC was significantly inhibited in the spleen spontaneous metastasis model (Fig. 5b–g). These findings demonstrate that hepatocyte-derived host VEGF, but not tumour cell-derived VEGF is crucially required for facilitating liver metastasis.

### Off-drug promotes metastasis in a CRC orthotopic model

**Figure 6:**
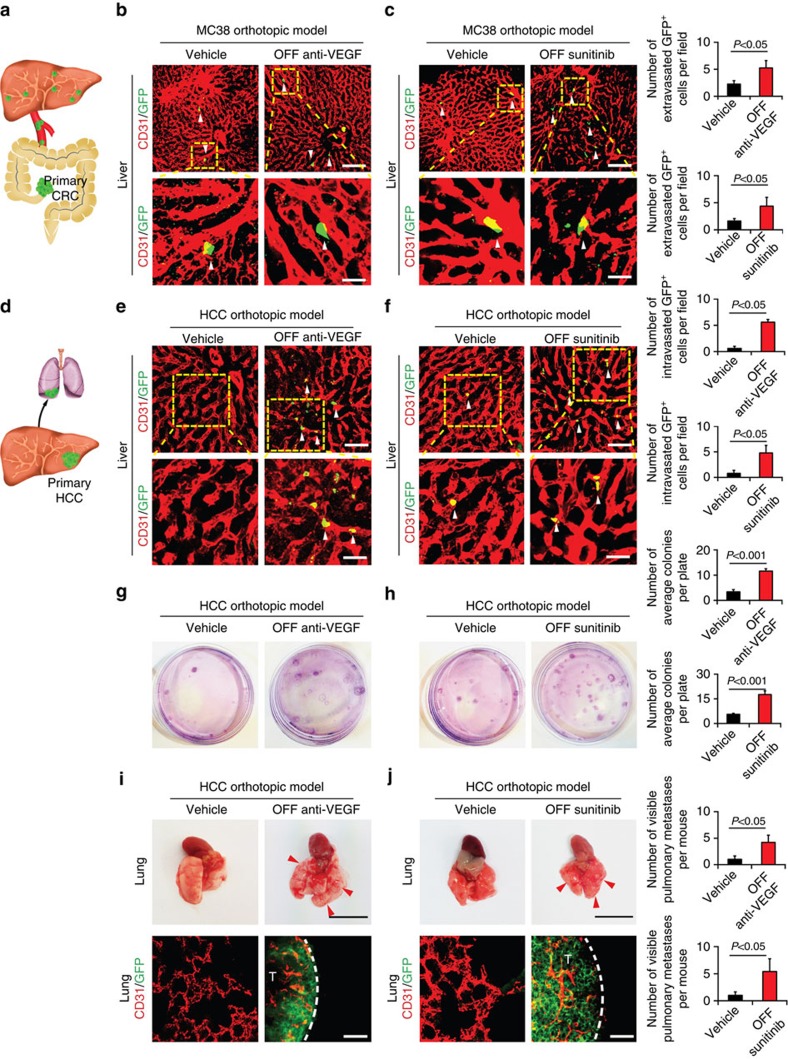
Extravasation and intravasation of tumour cells in orthotopic tumour models. (**a**) Schematic model of an orthotopic CRC metastasis model. CRC primary tumours were implanted in the caecum for subsequent liver metastasis. (**b**,**c**) Extravasation of CRC tumour cells at 24 h after drug cessation in various off-drug groups. Arrowheads point to extravasated tumour cells. Scale bar in upper panels, 100 μm. Scale bar in lower panels, 25 μm. Quantification of extravasated GFP^+^ tumour cells (*n*=8 fields per group). (**d**) Schematic model of HCC tumour cell implantation in liver and lung metastasis. (**e**,**f**) Intravasation of HCC tumour cells at 24 h after tumour cell implantation in various off-drug groups. Arrowheads point to extravasated tumour cells. Scale bar in upper panels, 50 μm. Scale bar in lower panels, 25 μm. Quantification of intravasated GFP^+^ tumour cells (*n*=8 fields per group). (**g**,**h**) Representative circulating tumour cell colony pictures and quantification of visible circulating tumour cell colonies (*n*=6 animals per group). (**i**,**j**) Representative lung pictures and quantification of visible metastatic nodules (*n*=6 animals per group). Red arrowheads point to visible metastatic nodules. Immunohistological analysis of lung metastasis. Dashed lines mark the borders between tumour and lung tissues. T, tumour; Scale bar in upper panels, 1 cm. Scale bar in lower panels, 50 μm. OFF, off-drug; ON, on-drug. (mean±s.e.m., Student's *t*-test).

To recapitulate the clinical situation of CRC metastasis, we further developed an orthotopic model of cancer metastasis by implanting CRC tumour cells in the caecum of the mouse colon (Fig. 6a). CRC tumour cells were labelled with GFP for monitoring metastasis. In this model, tumours were implanted to caecum before receiving drug therapy. When tumours reached an average size of ∼1.0 cm (ref. 3), tumour-bearing mice were systemically treated with VEGF blockade and sunitinib until tumour reached to ∼1.5 cm^3^. At day 6 after withdrawal of VEGF blockade and at day 2 after withdrawal of sunitinib, mice were killed for immunohistological examination of tumour cell extravasation. At these time points, tumour angiogenesis was significantly inhibited (Supplementary Fig. 7a,b). In both VEGF blockade and sunitinib off-drug groups, significantly increased extravasation of tumour cells were observed in liver tissues (Fig. 6b,c). Therefore, discontinuation of antiangiogenic therapy promotes cancer metastasis in an orthotopic primary tumour model.

### Off-drug promotes lung metastasis in a HCC orthotopic model

In addition to increases of tumour cell extravasation, discontinuation of anti-VEGF therapy-triggered hepatic microvascular alterations could also potentially promote hepatic cellular carcinoma (HCC) metastasis by a mechanism of facilitating tumour cell intravasation. To test this possibility, Hepa1-6 tumour cells were implanted into liver tissues at day 6 off-VEGF blockade and at day 2 off-sunitinib therapy. After 24 h off-drugs, tumour cell intravasation in the same liver lobe, but apart from the primary site, was subjected for immunohistochemical analysis. Indeed, higher numbers of intravasated tumour cells were present in off-antiangiogenic drug-treated groups relative to their respective controls (Fig. 6d–f). Circulating tumour cells were detected by culturing tumour cells in the peripheral blood. Significant increases of tumour cell colonies were detected in groups receiving discontinuation of anti-VEGF therapy (Fig. 6g,h). Tumour-bearing mice were killed ∼4 weeks after tumour cell implantation and liver weight was not significantly different from the control group (Supplementary Fig. 7c). However, significant increases of metastatic nodules were observed in lung tissues, suggesting increase of HCC intravasation in the liver microvasculature (Fig. 6i,j). Taken together, these data show that discontinuation of anti-VEGF therapy promotes HCC metastasis through a possible mechanism of enhancing intravasation via hyper-permeable liver microvasculatures.

## Discussion

Based on the antiangiogenic concept for cancer therapy, antiangiogenic drugs should be sustainably delivered to cancer patients as non-stop treatment. Discontinuation of antiangiogenic therapy would cause rapid revascularization in tumours and perhaps even a ‘rebound effect' of tumour angiogenesis^40^. One of the mechanisms underlying rapid revascularization is that antiangiogenic treatment triggers tumour hypoxia, which induces expression of hypoxia-regulated angiogenic factors such as VEGF (ref. 41). In the present study, we have found that off-targets of antiangiogenic therapy induce hypoxia and VEGF expression in healthy tissues such as in the liver. Like endocrine organs in the body, homeostasis of liver sinusoidal vasculatures is maintained by VEGF (ref. 42). Systemic inhibition of VEGF functions by drugs such as bevacizumab and sunitinib would block the physiological functions of VEGF in the liver, leading to marked regression of microvessels^32^,^33^. It seems that the regeneration mechanism of these sinusoidal vasculatures on cessation of treatment is also dependent on the availability of VEGF. Our findings validate the previous notion that vascular regeneration occurs along the regressive trail of the basement membrane-constituted sleeves that retained expression of collagen IV (ref. 33).

An interesting notion is that the off-drug-triggered liver revascularization exhibited a transient hyper-leakiness and the regenerated endothelium contains enlarged open-pores that were not seen under physiological conditions. Some of these enlarged open pores have sizes of ∼1 μm in diameters. Along with these structural changes, expression of VE-cadherin, which is crucial for maintenance of inter-endothelial cell tight junctions^43^,^44^ is also lost. Thus, hyper-vascular permeability is likely achieved by enlargement of fenestrated open-pores in endothelium and opening of inter-endothelial cell tight junctions. Would these structural changes permit tumour cell intravasation and extravasation? In different metastasis models, we show that marked increases of tumour cell extravasation through the wall of regenerated liver microvasculatures. Given the fact that some open-pore sizes reach 1 μm in diameter and tumour cell possesses the intrinsic features of spindle-like morphology, it is highly plausible for tumour cells to transmigrate through these large open pores. In particular, metastatic tumour cells undergo epithelial-mesenchymal transition^45^, exhibiting highly elongated spindle-like morphology that might potentially migrate through the altered endothelium. Alternatively, a piggyback of tumour cell transmigration mechanism with aides of inflammatory cells or stromal fibroblasts might also exist as these stromal cells are known to facilitate cancer metastasis^46^,^47^,^48^. The loss of VE-cadherin supports the opening of inter-endothelial cell junction and transmigration of tumour cell through the vessel wall.

One of the surprising findings of our study is that discontinuation of antiangiogenic therapy created a long-lasting effect of sinusoidal vascular changes. In both bevacizumab- and sunitinib-treated animals, after day 18 off-drug liver microvessel numbers recovered to the pre-treated levels and were superficially normalized. However, the endothelium contains high numbers of fenestrated open-pores and exhibits hyper-permeability. Although off-anti-VEGF therapy produces long-lasting impacts on liver vascular structures, discontinuation of VEGF blockade seems to produce longer sinusoidal dilation than sunitinib. The disparity between anti-VEGF neutralizing antibody and sunitinib perhaps reflects the difference of pharmacokinetics of these two drugs. Even though the anti-VEGF antibody has a long half-life that is equivalent to bevacizumab, withdrawal of the antibody produced a rapid revascularization in the liver, which recovers to the untreated level within 12 days. The antibody molecules are still present in the body as antigen–antibody complexes that lack ability for VEGF neutralization. Functional inactivation of anti-VEGF antibody also supports the fact of existence of high levels of biologically active VEGF in the tumour microenvironment after cessation of anti-VEGF therapy. There are probably two mechanisms involving in off-anti-VEGF therapy-triggered long-lasting liver vascular changes. First, remodelling of vascular fenestrations and perhaps tight junctions needs longer time than growing new vessels. Second, induction of sinusoidal dilations and other structural changes are likely more sensitive to VEGF than angiogenesis. Slight increases of VEGF molecules after cessation of anti-VEGF drugs are still able to maintain hyper-permeability of liver sinusoidal vasculatures.

Prolonged hyper-leakiness of liver microvasculatures after discontinuation of anti-VEGF therapy provides greater chances for tumour cell intravasation and extravasation through the vessel wall. Tumour cell extravasation through leaky liver microvessels occurs within a relatively short time. After 24 h tumour cell implantation, significant numbers of circulating tumour cells were extravasated in the liver tissue. This finding suggests that persistent tumour cell extravasation occurs in the liver tissue after discontinuation of antiangiogenic therapy. This is a clinically important issue because a primary tumour continuously release tumour cells into the circulation^49^. Circulating tumour cells disseminated from the primary site would serve as an incessant source for extravasation. In a clinically relevant orthotopic CRC model in which spontaneous cancer metastasis occurs, we have observed significant increases of tumour cell extravasation in livers after cessation of antiangiogenic therapy. Highly leaky liver microvessels also facilitate intravasation of tumour cells in an orthotopic HCC model.

During clinical practice with antiangiogenic cancer therapy, it is almost inevitable for discontinuation of antiangiogenic therapy owing to ineffectiveness, development of drug resistance, adverse effects and economically unaffordable high costs. Decisions of continuation and discontinuation of antiangiogenic drugs are often rendered based on therapeutic efficacy by monitoring primary tumour changes. The consequences of discontinuation of treatment in facilitating cancer metastasis in other organs are completely overlooked. Another challenging issue in relation to discontinuation of antiangiogenic therapy is the current drug delivery scheduling. For treatment of cancer patients, bevacizumab (once injection per month) and for subnitinib (4 week on and 2 week off) were systemically administrated in a disruptive scheduling. Would these scheduling increase cancer metastasis through mechanisms described in our study? Although there is no definite answer to this important clinical question, we can reasonably speculate that it is highly plausible for current antiangiogenic therapy scheduling to promote cancer metastasis. What is optimized scheduling of antiangiogenic therapy for human cancer patients? One possible solution would be to administrate low-dose of antiangiogenic drugs as non-stop maintenance therapy. If so, survival benefits of antiangiogenic therapy could be potentially improved in cancer patients. This interesting issue warrants clinical validation.

Our present data provide new mechanistic insights on withdrawal of antiangiogenic drug-induced liver cancer metastasis. As bevacizumab is used as the first-line therapy for treatment of CRCs that often metastasize to liver tissues, our findings are clinically relevant. In support of our results, clinical trial data using prolonged and sustained antiangiogenic regimens demonstrate beneficial improvement compared with short-term therapy. Based on these preclinical and clinical findings, we reasonably propose non-stop antiangiogenic therapy should be considered in cancer patients. Rigorous clinical trials by monitoring cancer metastasis after discontinuation of antiangiogenic therapy warrant further consideration.

## Methods

### Cell culture

VEGF-null 528ras tumour cell line was kindly provided by Dr Janusz Rak at the McGill University, Montreal, Canada; Mouse MC38 colon cancer cell line was kindly provided by Dr Rubén Hernández at the Gene Therapy Unit, Center for Applied Medical Research, University of Navarra, Pamplona, Navarra, Spain; and Hepa 1–6 cell line was kindly provided by Dr Takuji Torimura at the Division of Gastroenterology, Department of Medicine, Kurume University School of Medicine, Kurume, Fukuoka, Japan. VEGF-null 528ras fibrosarcoma, MC38 colon cancer and HCC Hepa 1–6 cells were cultured in DMEM (HyClone; Cat. No. SH30243.01) supplemented with 10% (vol/vol) heat-inactivated FBS (HyClone; Cat. No. SH30160.03), 100 U ml^−1^ penicillin, and 100 μg ml^−1^ streptomycin (HyClone; Cat. No. SV30010). MC38, Hepa 1-6 and 528ras cells were stably transfected with luciferase or *Egfp* reporter genes, followed by sorting with FACS. All the cell lines were not authenticated after purchase or transferred from other laboratories. We routinely tested mycoplasma contaminations in all our cell lines and they were negative.

### Animals

All animal studies were approved by the Animal Ethics Committee of Northern Stockholm. Female C57Bl/6 mice at age 6–8 weeks were provided by the MTC animal facility of the Karolinska Institutet, Sweden, and caged in a group of 10 or fewer mice per cage. *Vegf*^*flox/flox*^ mice in C57Bl/6 background were kindly provided by Dr Napoleone Ferrara from Genentech Inc., San Francisco, USA; and immunodeficient *CB17/Icr-Prkdcscid/IcrCrl* mice were purchased from the Charles River Laboratories.

### Orthotopic tumour and metastasis models

Before tumour implantation, mice were anaesthetized with hypnorm-dormicum (Vetapharma, Leeds, UK) at 1:1 ratio. For spleen tumour implantation, a left subcostal surgical incision was created and 1 × 10^6^ tumour cells in 30 μl were injected into the exposed hemispleen of each mouse. For liver portal vein tumour model, 1 × 10^6^ tumour cells in 30 μl were injected into the portal vein of each mouse. For the orthotopic colon cancer model, 1 × 10^6^ MC38 colon cancer cells were injected into the caecum wall using a 33-gauge Hamilton syringe (Cat. No. 7803-05, Hamilton). For the liver orthotopic HCC model, 2 × 10^6^ HCC Hepa 1–6 cells in 30 μl were injected into the left lobe of each mouse liver. Peritoneum and skin were surgically sutured with a 4-0 running stitch (Cat. No. V422H, Ethicon) and mice were simultaneously treated with a pain killer (Temgesic (Reckitt Benckiser, Berkshire, UK), 0.1 mg kg^−1^, twice per day) for 2 consecutive days after operation. In some experiments, primary tumours and spleens were surgically removed under an anesthesthetic condition. Approximately 2–3 weeks after tumour implantation, hepatic metastases were detected using an *in vivo* imaging system. Between 2 and 4 weeks after tumour implantation, mice were killed and liver or lung tissues were analysed for visible tumour nodules and histologically examined. In all experiments, 6–12 mice were used in each group.

### Anti-VEGF treatment

A humanized rabbit anti-mouse monoclonal VEGF-specific neutralizing antibody (BD0801) was kindly provided by the Simcere Pharmaceutical Company (Nanjing, Jiangsu, China) 36,50. A non-immune rabbit IgG isotype (Cat. No. 10500C, Invitrogen) was used as a control vehicle. Sunitinib was purchased from the LC laboratory (Cat. No. S-8803, LC Laboratories) and was diluted with drinking water. Anti-VEGF neutralizing antibody at the dose of 5 mg per kg was intraperitoneally injected twice per week into each mouse. Sunitinib was orally administrated once daily at the dose of 100 mg kg^−1^. Tissue samples were collected at days 0, 3, and 7 and off-drug at days 2, 4, 6, 8, 10, 12, 14 and 18. For scanning electron microscopy analysis, additional time points including off-drug days 21, 28 and 42 were used. For cancer metastasis models, similar treatment schedules were used before or after tumour implantation. At day 6 off-drug, animals of anti-VEGF-treated and vehicle-treated groups were examined. For sunitinib-treated and vehicle-treated groups, animals at day 2 off-drug were examined.

### Immunoblot

Fresh liver tissues were immediately lyzed in the presence of proteinase and phosphatase inhibitors (Cat. No. P8340, Sigma; 1:100; Cat. No. 5870, Cell Signaling; 1:100). An equal amount of protein samples from each group was separated by a 4–12% Bis-Tris NuPAGE gradient gel, followed by transferring onto a nitrocellulose membrane. Membranes were probed overnight at 4 °C with a rabbit anti-phospho-VEGFR2 antibody (Cat. No. 2471, Cell Signaling; 1:1,000), a rabbit anti-total-VEGFR2 antibody (Cat. No. 2479, Cell Signaling; 1:1,000), and a mouse anti-β-actin antibody (Cat. No. 3700, Cell Signaling; 1:1,000) in PBS with 5% BSA containing 0.1% Tween 20. Membranes were incubated at room temperature for 1 h with a donkey anti-rabbit IgG antibody (Cat. No. 926-68073, IRDye 680RD; LI-COR; 1:1,5000) and a donkey anti-mouse IgG antibody (Cat. No. 926-32212, IRDye 800CW; LI-COR; 1:1,5000). Protein bands were visualized and quantified using the ODYSSEY CLx (LI-COR) detection system at 700 and 800 nm wavelengths.

### RT-PCR and qPCR

PCR with reverse transcription (RT–PCR) and quantitative PCR (qPCR) were performed according to standard protocols^51^,^52^,^53^. In brief, total RNA samples were prepared by a GeneJet RNA Purification Kit (Cat. No. K0731, Thermo Scientific, MA, USA), and cDNA was synthesized using a RevertAid H minus First Strand cDNA Synthesis Kit (Cat. No. K1632, Thermo Scientific, MA, USA). RT-PCR was performed using a DreamTaq Green PCR Master Mix Kit (Cat. No. K1082, Thermo Scientific, MA, USA) and 2720 PCR machine (Applied Biosystems, CA, USA). qPCR was performed using a Power SYBR Green Master Mix Kit (Cat. No. 4367659, Applied Biosystems, CA, USA) and a Step One Plus real-time PCR system (Applied Biosystems, CA, USA). The following primers were used: mouse *Gapdh* forward: 5′- CCAGCAAGGACACTGAGCAA -3′ and mouse *Gapdh* reverse 5′- GGGATGGAAATTGTGAGGGA -3′; mouse *Hif1α* forward: 5′- GTCGGACAGCCTCACCAAACAG -3′ and mouse *Hif1α* reverse 5′- TAGGTAGTGAGCCACCAGTGTCC -3′. Triplicate samples were analysed in each group.

### ELISA

Fifty milligrams of fresh liver or tumour tissue were homogenized in a 500 μl lysis buffer (Cat. No. C3228, Sigma) containing a cocktail of proteinase inhibitors (Cat. No. P8340, Sigma) using an electronic homogenizer. For tumour cells, 1 × 10^6^ cells were lyzed with the same lysis buffer. Tissue homogenates and lyzed cells were centrifuged at 10,000*g* for 15 min and 50 μl of supernatant from each sample were analysed using an enzyme-linked immunosorbent assay (ELISA) kit detecting mouse VEGF (Cat. No. MMV00, R&D Systems Inc.) according to the manufacturer´s instruction. For detection of non-bound free VEGF, tissue supernatants were pre-treated with Protein A/G Sepharose beads (Cat. No. sc-2003, Santa Cruz) to remove VEGF-A that bound to the neutralizing antibody. The antibody-free tissue samples were subsequently used for the VEGF-ELISA detection.

### Immunohistochemistry and whole-mount staining

Paraffin-embedded tissues were cut in 5-μm-thickness, mounted onto glass slides, baked for one hour at 60°C deparaffinized in Tissue-Clear (Cat. No. 1466, Sakura), and sequentially rehydrated in 99%, 95% and 70% ethanol. Tissue slides were counterstained Haematoxylin and Eosin before dehydration with 95 and 99% ethanol and were mounted with PERTEX (Cat. No. 00801, HistoLab). Stained tissues were analysed under a light microscope (Nikon Eclipse TS100). Whole-mount staining was performed using standard protocol^54^,^55^,^56^,^57^. Briefly, small pieces of tissues were cut into thin slices and fixed in 4% PFA overnight, followed by treatment with proteinase K (20 μg ml^−1^). Tissues were incubated overnight at 4 °C with primary antibodies: a goat anti-mouse CD31 antibody (Cat. No. AF3628, R&D); a rabbit anti-mouse VE-cadherin antibody (Cat. No. ab33168, Abcam); a rabbit anti-mouse Fibronectin antibody (Cat. No. ab23750, Abcam); a rabbit anti-mouse Collagen IV antibody (Cat. No. ab19808, Abcam); a rabbit anti-mouse Laminin antibody (Cat. No. ab30320, Abcam); a rabbit anti-mouse NG2 antibody (Cat. No. MAB5384, Millipore); and a rabbit anti-mouse GFP antibody (Cat. No. A11122, Invitrogen). After rigorous washing, tissue samples were further stained for 2 h at room temperature with secondary antibodies to recognize their respective primary antibodies: a donkey anti-goat Alexa 555 antibody (Cat. No. A21432, Invitrogen); a donkey anti-goat Alexa 488 antibody (Cat. No. A11055, Invitrogen); a donkey anti-goat Alexa 647 antibody (Cat. No. A21447, Invitrogen); and a donkey anti-rabbit Alexa 555 antibody (Cat. No. A31572, Invitrogen). After thorough washing, slides were mounted and examined under a confocal microscope (Zeiss Confocal LSM510 Microscope). Three-dimensional images of each tissue sample were projected and quantitative analyses from at least eight random different tissue sections were performed using an Adobe Photoshop CS software programme.

### Blood perfusion and vascular permeability

At different time points after treatment, each mouse was anaesthetized and i.v. injected with 100 μl of 2,000-kDa-lysinated fluorescein-labelled dextran (Cat. No. D7139, Invitrogen)^37^. Animals were killed 5 min after dextran injection, and tissues were dissected and immediately fixed with 4% PFA at 4 °C. For permeability assay, 100 μl of 70-kDa-lysinated fluorescein-labelled dextran (Cat. No. D1818, Invitrogen) was i.v. injected into each mouse. Animals were killed 15 min after dextran injection. Liver tissues were carefully dissected, whole-mount stained and examined by confocal microscopy.

### Scanning electron microscopy

At different time points after treatment, animals were killed and immediately fixed by vascular perfusion with 2.5% (vol/vol) glutaraldehyde plus 1% PFA in 0.1 M phosphate buffer (pH 7.4). Liver tissues were dissected and further fixed with the same fixative. After rinsing with distilled water, samples were dehydrated with a stepwise ethanol gradient and placed in acetone. Specimens were then dried using a critical point dryer (Balzer, CPD 010, Lichtenstein) with carbon dioxide. After drying, specimens were mounted on an aluminium stub and coated with Platinum (Bal-Tec SCD 005). Tissue specimens were analysed under an Ultra 55 field emission scanning electron microscope (Zeiss, Oberkochen, Germany) at 3 kV (ref. 58).

### Vascular casting

Vascular casting was performed according to our standard protocol^59^. In brief, mice were anaesthetized with hypnorm-dormicum (Vetapharma, Leeds, UK) at 1:1 ratio. Mice were surgically operated to expose the aorta arch. A closed i.v. catheter system (Cat. No. 383532, BD Biosciences) connected to a reservoir of physiological saline (0.9% NaCl) was inserted into the aorta. Mice were flushed with 10–20 ml Ringer's buffer, and subsequently injected with a 10–20 ml mixture solution containing PU4ii resin (VasQTec, Zurich, Switzerland), ethyl methyl ketone (10:4 dilution; Cat. No. 1060141000, Millipore) and hardener (VasQTec, Zurich, Switzerland). Mice were kept overnight at room temperature to allow resin polymerization. Hepatic tissues were dissected and treated with one or more changes of 20% NaOH. Casts were rinsed first in distilled water, then in 96% ethanol and dried in a desiccator for 48 h. Relevant pieces of cast were dissected and mounted onto aluminium stubs using double-sided carbon tape. Preparations were sputter coated with 4 nm gold on a low vacuum coater (EM ACE200, Leica Microsystems GmbH, Wetzlar, Germany), and viewed in a XL30 scanning electron microscope (FEI, OR, USA).

### Adenovirus delivery

Adenovirus-Cre was purchased from Vector Biolabs (Cat. No.1045, Vector Biolabs). Adenovirus stocks were prepared according to our standard protocol^54^. Each mouse was i.v. injected on every 5th day with 100 μl of adenovirus containing 1 × 10^9^ PFU particles.

### *In vivo* bioluminescent imaging

Primary and metastatic tumour masses were monitored with an IVIS Spectrum CT system (PerkinElmer). Briefly, tumour-bearing mice were injected with D-luciferin (150 mg kg^−1^, PerkinElmer) and luminescence-positive signals were detected by IVIS Spectrum CT system after 10–20 min injection (PerkinElmer). For some experiments, dissected liver tissues were subjected for imaging analysis. Metastatic lesions were further validated by H&E histological analysis and fluorescent microscopy.

### Statistical analysis

For quantitative analysis, randomized micrographs from at least eight different fields were used. The Adobe Photoshop CS4 software programme was used with a colour range tool and a count tool to detect positive areas and numbers. Sample sizes were carefully chosen for each experiment based on pilot experiment examinations and sufficient statistic powers. For all tumour studies, at least six animals per group were used to ensure the adequate power. Each experiment was repeated 2 times. Animals were excluded from the analysis if they did not meet the pre-established criteria of the Karolinska Institute template. In all animal experiments, experimental animals were randomly and blindly divided into each group to receive various treatments. A standard two-tailed Student's *t*-test was used for all statistical analyses. All sample sizes were appropriate for assumption of normal distribution and variance was similar between compared groups. The statistical values of *P*<0.05, *P*<0.01 and *P*<0.001 were considered statistically significant. Values of mean determinants are presented as±s.e.m. The authors declare that the data supporting the findings of this study are available within the article and its Supplementary Information files.

### Data availability

The authors declare that the data supporting the findings of this study are available within the article and its Supplementary Information files.

## Additional information

**How to cite this article**: Yang, Y. *et al*. Discontinuation of anti-VEGF cancer therapy promotes metastasis through a liver revascularization mechanism. *Nat. Commun.* 7:12680 doi: 10.1038/ncomms12680 (2016).

## Supplementary Material

Supplementary Information Supplementary Figures 1-8
