# Supplementary material for: Discontinuation of anti-VEGF cancer therapy promotes metastasis through a liver revascularization mechanism
Source: Nat Commun. 2016 Sep 1;7:12680. doi: 10.1038/ncomms12680 (PMC5025794; doi:10.1038/ncomms12680)
Supplement: Supplementary Information — Supplementary Figures 1-8 [file ncomms12680-s1.pdf]

Supplementary Figure and Figure Legends

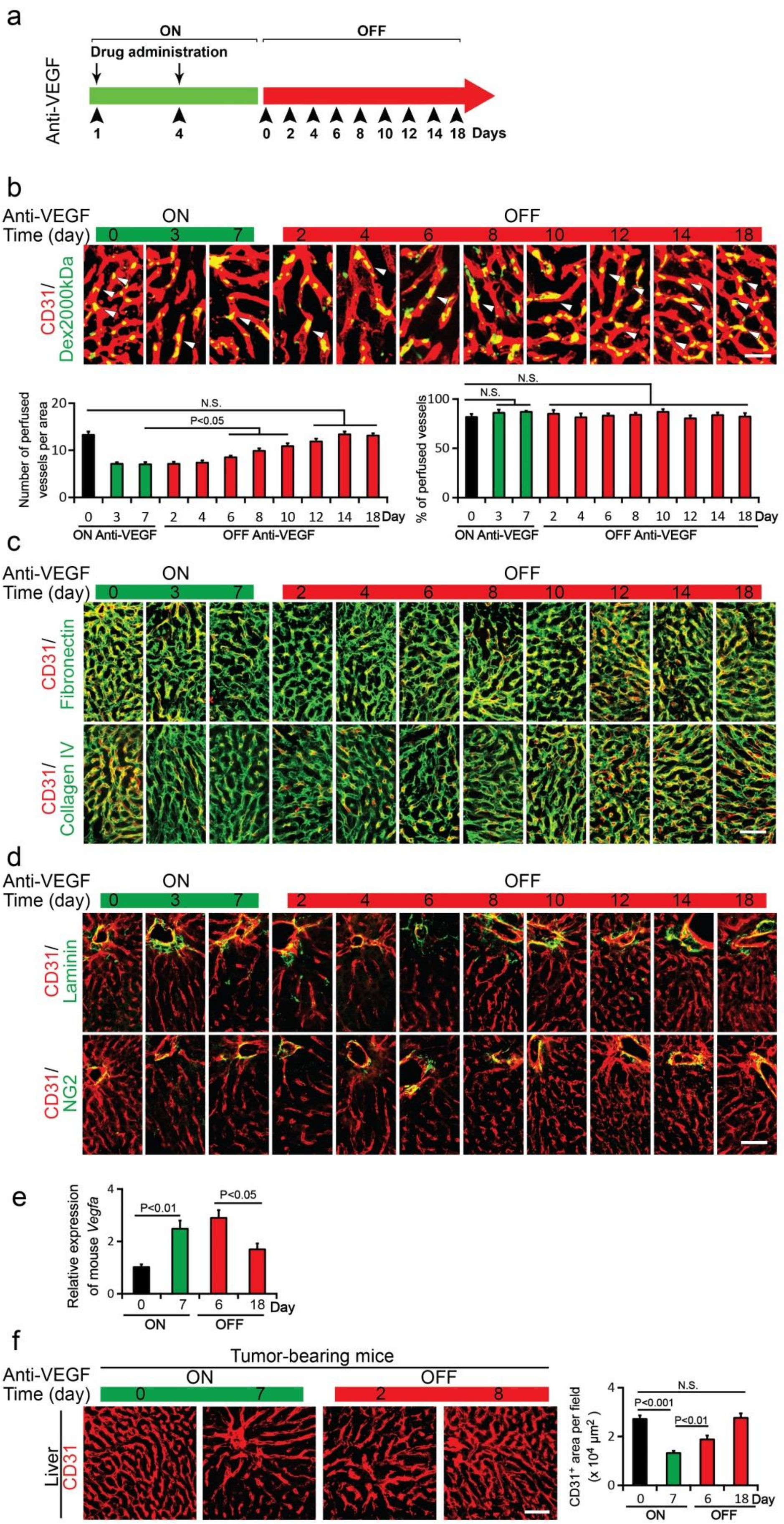
a
ON
OFF
Drug administration
Anti-VEGF
1
4
0 2 4 6 8 10 12 14 18 Days
b
Anti-VEGF
Time (day)
ON
OFF
0 3 7
2 4 6 8 10 12 14 18
CD31/
Dex2000kDa
Number of perfused vessels per area
N.S.
P<0.05
0 3 7 2 4 6 8 10 12 14 18 Day
ON Anti-VEGF
OFF Anti-VEGF
% of perfused vessels
N.S.
N.S.
0 3 7 2 4 6 8 10 12 14 18 Day
ON Anti-VEGF
OFF Anti-VEGF
c
Anti-VEGF
Time (day)
ON
OFF
CD31/
Fibronectin
CD31/
Collagen IV
d
Anti-VEGF
Time (day)
ON
OFF
CD31/
Laminin
CD31/
NG2
e
Relative expression
of mouse Vegfa
P<0.01
P<0.05
0 7 6 18 Day
ON
OFF
f
Tumor-bearing mice
Anti-VEGF
Time (day)
ON
OFF
0 7 2 8
Liver
CD31
CD31+ area per field
(x 10^4 μm^2)
N.S.
P<0.001
P<0.01
0 7 6 18 Day
ON
OFF

Supplementary Figure 1: Vascular perfusion and expression of extracellular matrix proteins

(**a**) Schematic diagram of anti-VEGF treatment of withdrawal of drugs. Healthy mice were administrated with anti-VEGF antibody at day 1 and day 4. Liver tissues were examined at day 0, on-drug day 3 and day 7, and off-drug day 2, 4, 6, 8, 12, 14, 18. (**b**) Vascular perfusion of Fluorescein-labeled 2000-kDa-dextran in liver microvasculatures of various groups. Arrows point to dextran signals. Scale bar, 25 µm. Data were quantified from 9 random fields per group. (**c**) Immunohistochemical analysis of expression levels of fibronectin and collagen IV in various groups. Scale bar, 50 µm. (**d**) Immunohistochemical analysis of expression levels of laminin and NG2 in various groups. Scale bar, 50 µm. (**e**) Quantification of *Vegf* mRNA levels by qPCR (n = 3 samples per group). (**f**) $CD31^+$ liver microvessels in tumor-bearing mice received On and Off anti-VEGF treatment. Scale bar, 50 µm. Quantification of $CD31^+$ areas (n = 8 fields per group). ON = on-drug; OFF = off-drug. (mean ± s.e.m., N.S., Not significant, Student's *t*-test)

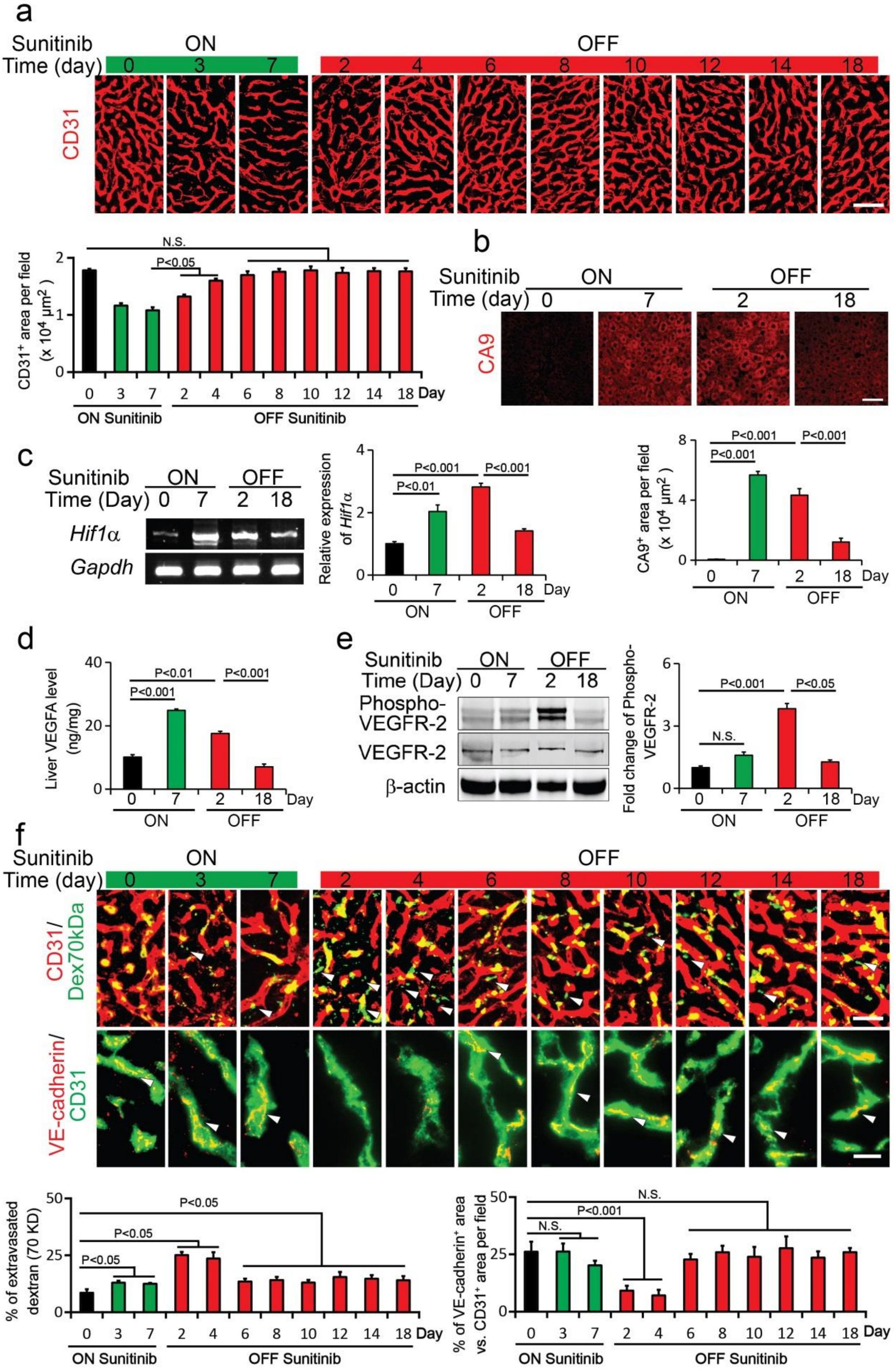
a
Sunitinib ON OFF
Time (day) 0 3 7 2 4 6 8 10 12 14 18
CD31
CD31+ area per field (x 10^4 μm^2)
N.S.
P<0.05
ON Sunitinib
OFF Sunitinib
Day
b
Sunitinib ON OFF
Time (day) 0 7 2 18
CA9
CA9+ area per field (x 10^4 μm^2)
P<0.001
c
Sunitinib ON OFF
Time (Day) 0 7 2 18
Hif1α
Gapdh
Relative expression of Hif1α
P<0.01
P<0.001
d
Liver VEGFA level (ng/mg)
P<0.001
P<0.01
e
Phospho-VEGFR-2
VEGFR-2
β-actin
Fold change of Phospho-VEGFR-2
P<0.05
f
CD31/ Dex70kDa
VE-cadherin/ CD31
% of extravasated dextran (70 KD)
% of VE-cadherin+ area vs. CD31+ area per field

Supplementary Figure 2. Revascularization of liver microvasculatures after withdrawal of systemic sunitinib treatment

(a) Time-course analysis of $CD31^+$ microvessels in livers receiving prior to sunitinib, on-drug, and off-drug. Scale bar, 50 µm. Data were quantified from 9 random fields per group. **(b)** Liver tissue hypoxia measured by CA9 expression in various groups. Scale bar, 100 µm. Data were quantified from 9 random fields per group. **(c)** RT-PCR and qPCR quantification of *Hif1a* expression in various on- and off-drug groups (triplicates per group). **(d)** ELISA measurement of liver VEGF in different groups (triplicates per group). **(e)** Measurement and quantification of total and phosphorylated VEGFR2 protein levels in various groups (triplicates per group). **(f)** Measurements of extravasation of Fluorescein-labeled 70-kDa-dextran and expression levels of VE-cadherin in different groups. Arrows point to leaked dextran signals. Scale bar in upper panel, 25 µm. Scale bar in lower panel, 10 µm. Data were quantified from 9 random fields per group. ON = on-drug; OFF = off-drug. (mean ± s.e.m., N.S., Not significant, Student's *t*-test).

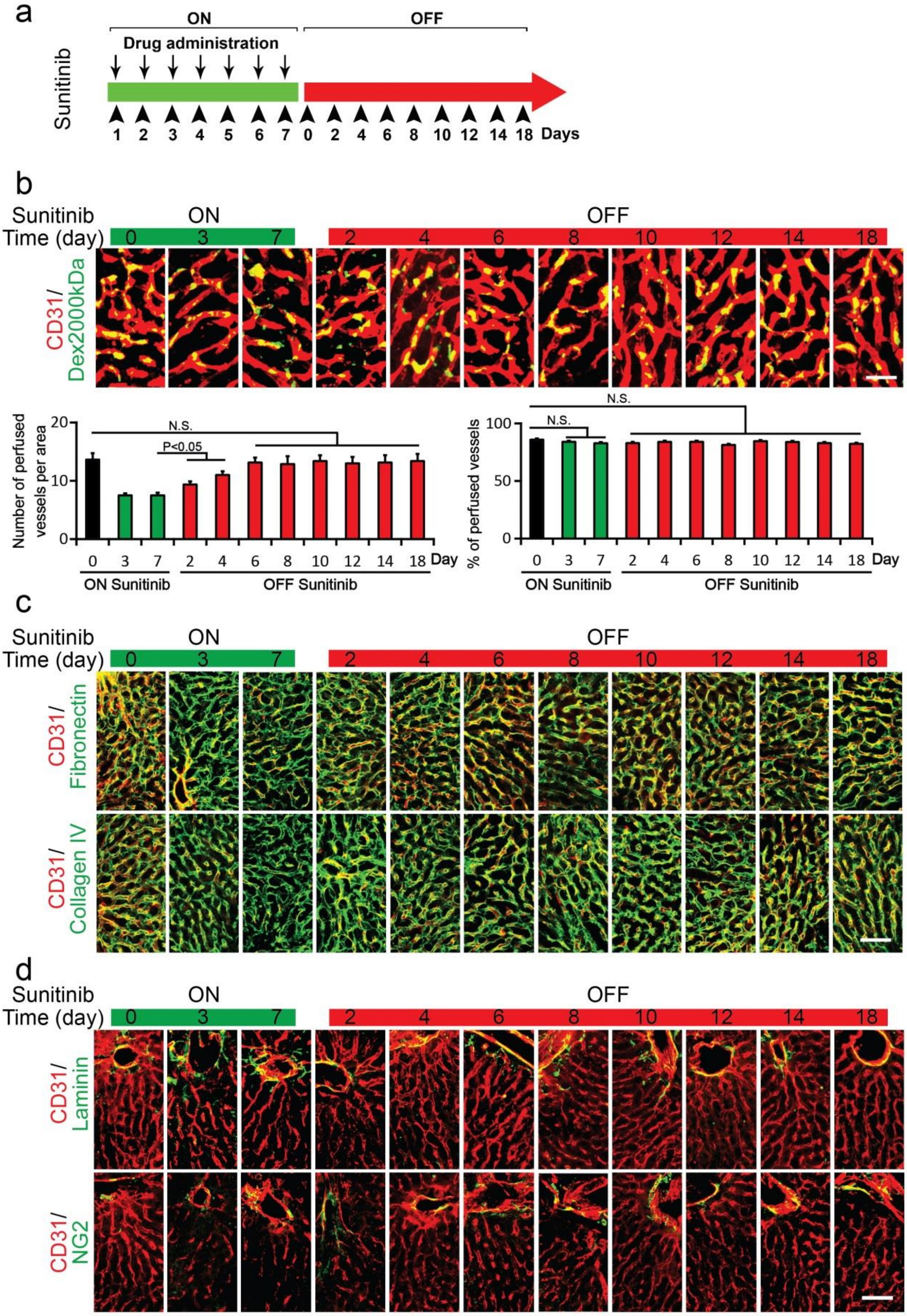

a
Sunitinib
ON
OFF
Drug administration
1 2 3 4 5 6 7 0 2 4 6 8 10 12 14 18 Days
b
Sunitinib
ON
OFF
Time (day)
0 3 7 2 4 6 8 10 12 14 18
CD31/ Dex2000kDa
Number of perfused vessels per area
N.S.
P<0.05
ON Sunitinib
OFF Sunitinib
Day
% of perfused vessels
c
CD31/ Fibronectin
CD31/ Collagen IV
d
CD31/ Laminin
CD31/ NG2

Supplementary Figure 3: Vascular perfusion and expression of extracellular matrix proteins in on- and off-drug-treated livers

(**a**) Schematic diagram of sunitinib treatment after withdrawal of drugs. (**b**) Vascular perfusion of Fluorescein-labeled 2000-kDa-dextran in liver microvasculatures of various groups. Arrows point to the dextran^{+} signals. Scale bar, 25 µm. Data were quantified from 9 random fields per group. (**c**) Immunohistochemical analysis of expression levels of fibronectin and collagen IV in various groups. Scale bar, 50 µm. (**d**) Immunohistochemical analysis of expression levels of laminin and NG2 in various groups. Scale bar, 50 µm. ON = on-drug; OFF = off-drug. (mean ± s.e.m., N.S., Not significant, Student's *t*-test).

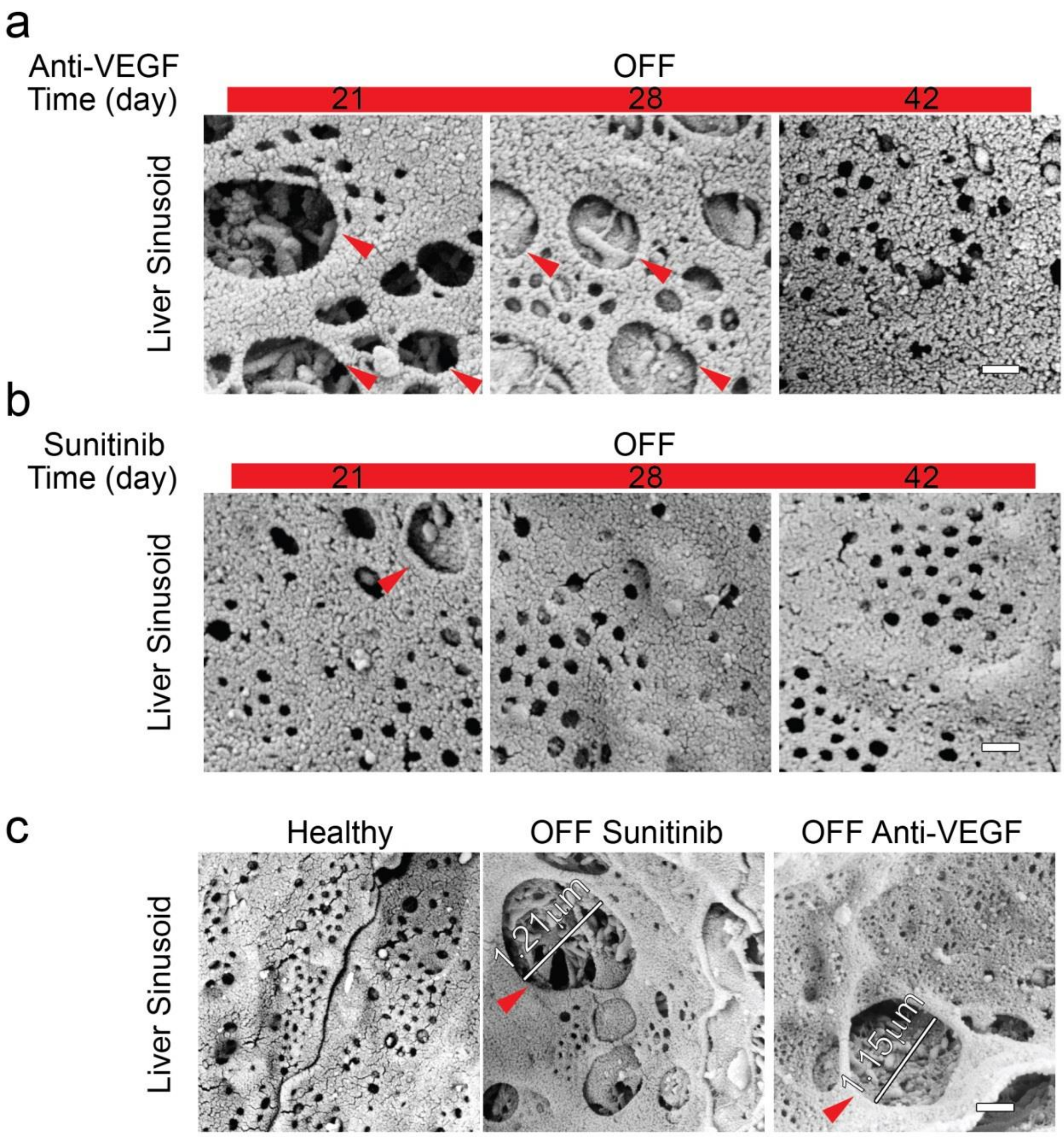

Supplementary Figure 4: SEM time-course analysis of sinusoidal liver microvessels

(**a, b**) Representative SEM micrographs of liver microvessel endothelium at different time points after cessation of VEGF blockade and sunitinib treatments. Red arrowheads point to enlarged open pores in the fenestrated endothelium. Scale bar, 200 nm. (**c**) Representative SEM micrographs of liver microvessel endothelium after cessation of VEGF blockade and sunitinib treatments. Red arrowheads point to biggest enlarged open pores in the fenestrated endothelium. Scale bar, 400 nm.

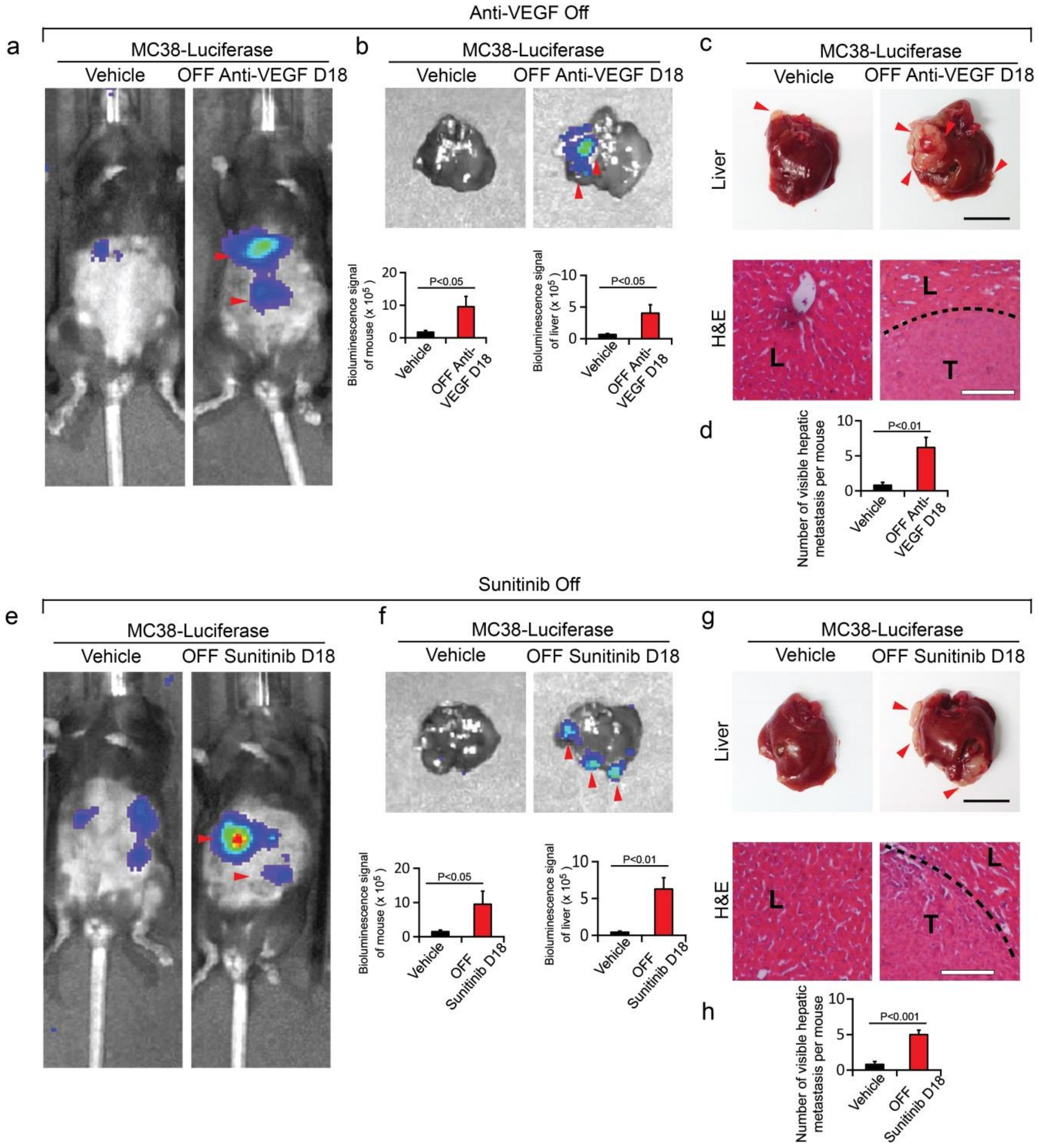

Supplementary Figure 5. Off-drug-promoted CRC tumor cell extravasation and metastasis at later time points

(**a, e**) Representative mouse pictures of various groups subjected to luminescent imaging analysis of luciferase activity in metastatic cancers. Red arrowheads point to luciferase positivity. Data were quantified from 6 mice per group. (**b, f**)

Representative liver micrographs subjected to luminescent luciferase activity analysis (n = 6 animals per group). Red arrowheads point to luciferase positivity. Quantifications of liver luciferase activity (n = 6 animals per group). (**c, g**) Representative liver pictures and H&E histological analysis of liver metastasis. Dashed lines mark the borders between tumor and liver tissues. T = tumor; L = liver. Scale bar in upper panel, 1 cm. Scale bar in lower panel, 100 µm. (**d, h**) Quantification of visible surface liver metastatic nodules (n = 6 animals per group). ON = on-drug; OFF = off-drug. (mean ± s.e.m., Student's *t*-test).

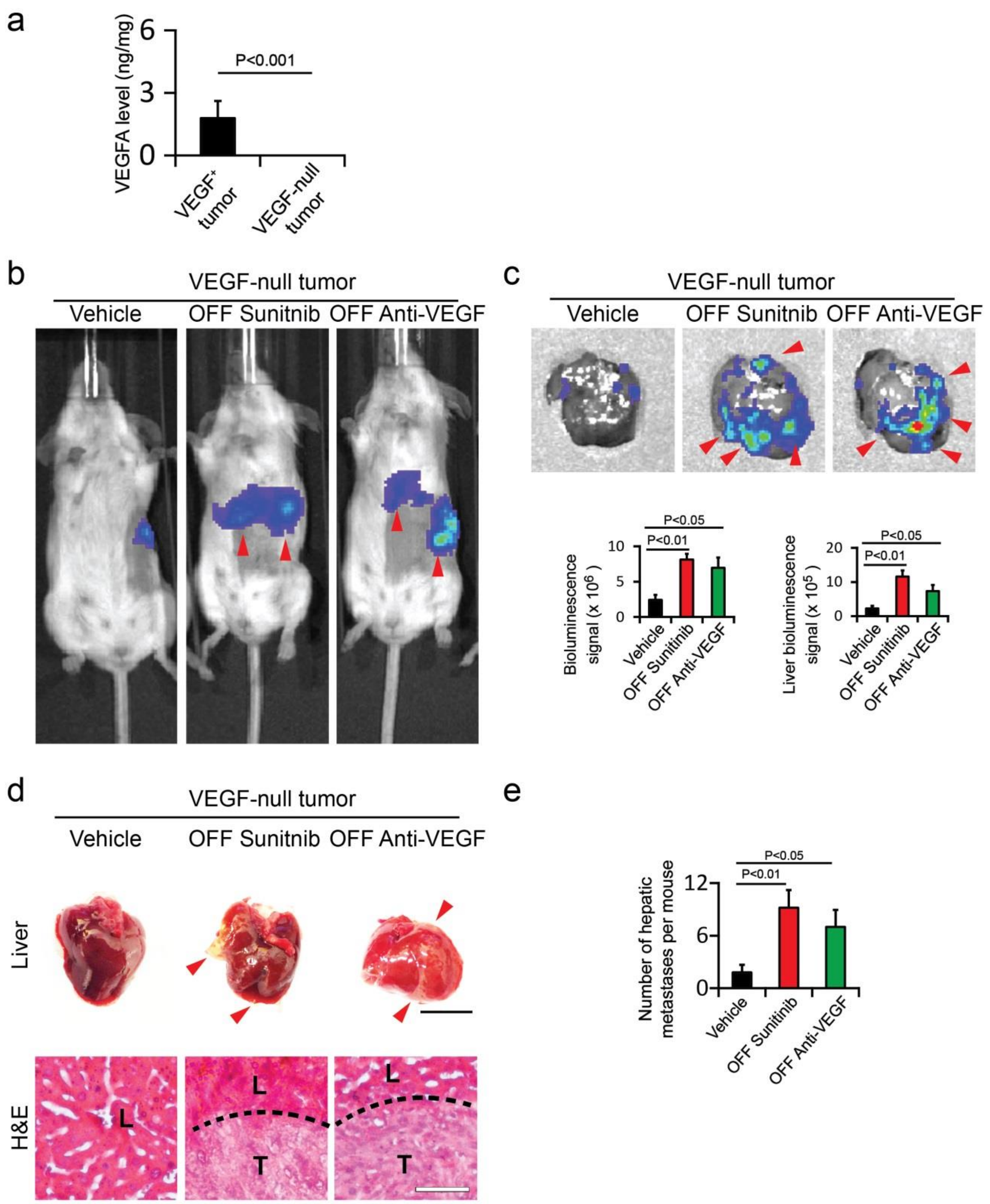

Supplementary Figure 6. VEGF-null tumor cell extravasation and metastasis in livers

(**a**) VEGF protein expression in VEGF$^+$ tumor and VEGF-null tumor cell lines (n = 3 samples per group). (**b**) Representative mouse pictures of various groups subjected to

luminescent imaging analysis of luciferase activity in metastatic cancers. Red arrowheads point to luciferase positivity. Data were quantified from 6 mice per group. (**c**) Representative liver micrographs subjected to luminescent luciferase activity analysis (n = 6 animals per group). Red arrowheads point to luciferase positivity. Quantification of liver luciferase activity (n = 6 animals per group). (**d**) Representative liver pictures and H&E histological analysis of liver metastasis. Dashed lines mark the borders between tumor and liver tissues. T = tumor; L = liver. Scale bar in upper panel, 1 cm. Scale bar in lower panel, 25 µm. (**e**) Quantifications of visible surface liver metastatic nodules (n = 6 animals per group). ON = on-drug; OFF = off-drug. (mean ± s.e.m., Student's *t*-test).

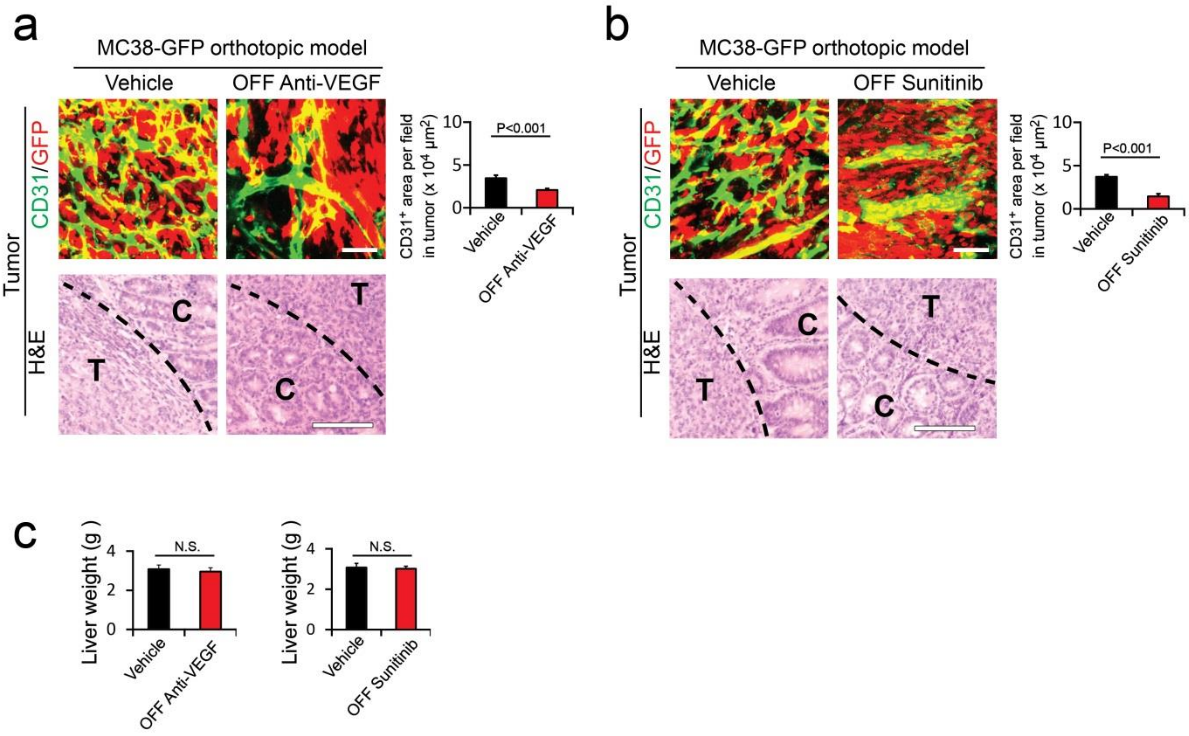

Supplementary Figure 7. Primary tumor in orthotopic CRC and HCC tumor models

(**a, b**) Immunohistological analysis of CRC primary tumors. Green: $CD31^+$ signals. Red: GFP^+ signals. H&E histological analysis of primary CRC tumors. Dashed lines mark the borders between tumor and cecum tissues. T = tumor; C = cyst. Scale bar in upper panel, 50 µm. Scale bar in lower panel, 100 µm. Quantification of $CD31^+$ area. (**c**) Quantification of liver weight after 4 weeks of HCC tumor implantation in an orthotopic HCC metastasis model. ON = on-drug; OFF = off-drug. (mean ± s.e.m., N.S., Not significant, Student's *t*-test).

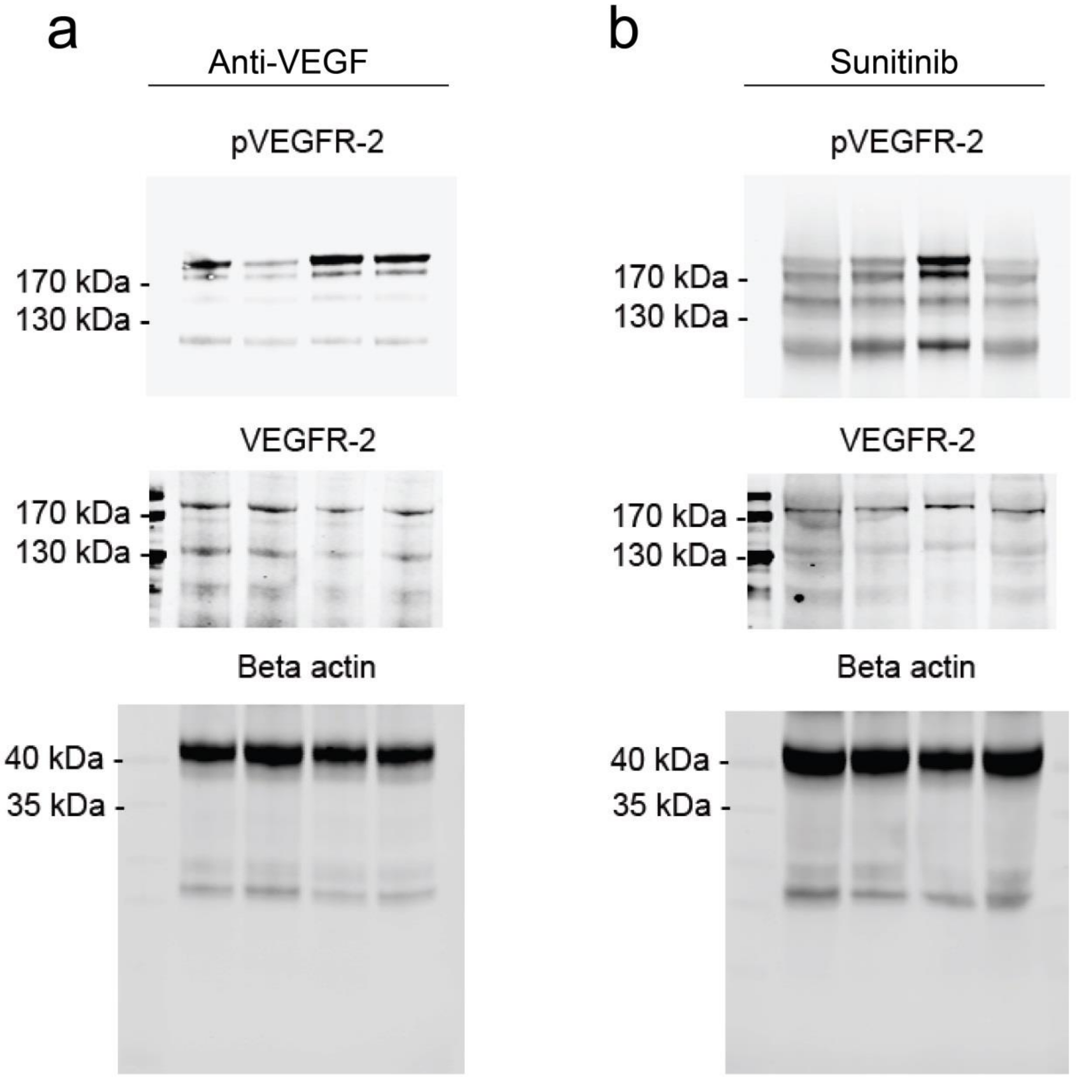

Supplementary Figure 8. Original full-length western blots

(**a**) Original western blots used for Fig. 1e. (**b**) Original western blots used for Supplementary Fig. 2e
